# In-Silico Prediction and Modeling of the Quorum Sensing LuxS Protein and Inhibition of AI-2 Biosynthesis in *Aeromonas hydrophila*

**DOI:** 10.3390/molecules23102627

**Published:** 2018-10-12

**Authors:** Farman Ali, Zujie Yao, Wanxin Li, Lina Sun, Wenxiong Lin, Xiangmin Lin

**Affiliations:** 1 Fujian Provincial Key Laboratory of Agroecological Processing and Safety Monitoring, College of Life Sciences, Fujian Agriculture and Forestry University, Fuzhou 35002, China; acrobat.web@gmail.com (F.A.); Mic_yao@live.com (Z.Y.); liwx2012@126.com (W.L.); sunlina19901220@163.com (L.S.); 2 Key Laboratory of Crop Ecology and Molecular Physiology (Fujian Agriculture and Forestry University) Fujian Province University, Fuzhou 35002, China

**Keywords:** *Aeromonas hydrophila*, LuxS, quorum sensing, 3Dstructure, i-TASSER, SAVES server, AI-2, molecular docking

## Abstract

*luxS* is conserved in several bacterial species, including *A. hydrophila,* which causes infections in prawn, fish, and shrimp, and is consequently a great risk to the aquaculture industry and public health. *luxS* plays a critical role in the biosynthesis of the autoinducer-2 (AI-2), which performs wide-ranging functions in bacterial communication, and especially in quorum sensing (QS). The prediction of a 3D structure of the QS-associated LuxS protein is thus essential to better understand and control *A. hydrophila* pathogenecity. Here, we predicted the structure of *A. hydrophila* LuxS and characterized it structurally and functionally with in silico methods. The predicted structure of LuxS provides a framework to develop more complete structural and functional insights and will aid the mitigation of *A. hydrophila* infection, and the development of novel drugs to control infections. In addition to modeling, the suitable inhibitor was identified by high through put screening (HTS) against drug like subset of ZINC database and inhibitor ((−)-Dimethyl 2,3-*O*-isopropylidene-l-tartrate) molecule was selected based on the best drug score. Molecular docking studies were performed to find out the best binding affinity between LuxS homologous or predicted model of LuxS protein for the ligand selection. Remarkably, this inhibitor molecule establishes agreeable interfaces with amino acid residues LYS 23, VAL 35, ILE76, and SER 90, which are found to play an essential role in inhibition mechanism. These predictions were suggesting that the proposed inhibitor molecule may be considered as drug candidates against AI-2 biosynthesis of *A. hydrophila.* Therefore, (−)-Dimethyl 2,3-*O*-isopropylidene-l-tartrate inhibitor molecule was studied to confirm its potency of AI-2 biosynthesis inhibition. The results shows that the inhibitor molecule had a better efficacy in AI-2 inhibition at 40 μM concentration, which was further validated using Western blotting at a protein expression level. The AI-2 bioluminescence assay showed that the decreased amount of AI-2 biosynthesis and downregulation of LuxS protein play an important role in the AI-2 inhibition. Lastly, these experiments were conducted with the supplementation of antibiotics via cocktail therapy of AI-2 inhibitor plus OXY antibiotics, in order to determine the possibility of novel cocktail drug treatments of *A. hydrophila* infection.

## 1. Introduction

*A. hydrophila* is a prevalent and opportunistic pathogen of aquatic organisms, including fish, shrimp, and prawns [1]. Furthermore, *A. hydrophila* also causes severe diseases in humans, including gastrointestinal illnesses, septicemia, and cellulitus [2,3,4]. In several bacterial species, gene expression is controlled by the secretion, detection, and production of extracellular signaling molecules (AI-2) that accumulate in environments in proportion to cell densities of signal molecule-producing cells. This phenomenon is commonly referred to as ‘quorum sensing’. The S-ribosyl-homocysteinase (LuxS) quorum-sensing (QS) system is found in several bacterial species [5] and was initially characterized as the regulator of bioluminescence in *Vibrio harveyi* [6]. QS has been well-studied due to its regulation of vital physiological mechanisms, including competence, sporulation, motility, biofilm formation, and its critical role in determining virulence. The presence of *luxS* homologs in both Gram-positive and Gram-negative bacteria suggests that AI-2 is a universal language for interspecies communication [7]. Moreover, LuxS is an integral component of the activated methyl cycle, which may also explain its widespread conservation [8].

LuxS encodes an enzyme that aids in the metabolism of S-adenosyl-methionine (SAM) and catalyzes the conversion of S-ribosyl-homocysteine (SRH) to homocysteine and 4,5-dihydroxy-2,3-pentanedione (4,5-DPD), in addition to several furanones [9,10,11]. Production of the autoinducer (AI-2) occurs when 4,5-DPD autocatalytically hydrolyzes [12]. The LuxS protein, S-ribosyl-homocysteinase, is associated with the synthesis of AI-2. AI-2 plays a crucial role in intercommunication among bacterial species that possess LuxS and that are at high cell densities [7,13]. The LuxS system has been thoroughly investigated in Gram-negative bacteria, which includes *A. hydrophila* and the marine *γ-proteobacterium, Vibrio harveyi* [14,15]. LuxP, which responds to AI-2 [16], has also been thoroughly studied in *V. harveyi*. However, bacteria without LuxP can also respond to AI-2, and other receptors can be involved in the biosynthesis of AI-2 precursors, including LsrB (*Bacillus, E. coli,* and *Salmonella enterica*) and rbsB in *H. influenza* [17]. However, the AI-2 receptor gene LuxP is prevalent in Gram-positive bacteria, although little is known regarding them. *luxS* is found in Gram-positive and Gram-negative bacteria, including *A. hydrophila*, and performs critical transcription functions in the regulation of genes associated with transport of nucleotides, metabolism, and the synthesis of cell walls or membranes [18]. In contrast, the deregulation mechanisms of those genes are still unknown. Furthermore, the LuxS system of *A. hydrophila* performs a vital role in in vitro biofilm synthesis. Mutant strains of *luxS* fail to produce mature biofilms [19], although increased concentrations of salinity and increased incubation periods also reduce biofilm formation, motility, protease biogenesis, and quorum sensing [20]. Moreover, mutation of the *luxS* AI-2 synthase gene decreases virulence towards hosts, although virulence can be recovered when AI-2 is added from exogenous sources [21].

LuxS belongs to the LuxS/MPP-like metallohydrolase superfamily of enzymes, according to the structural classification of proteins (SCOP) scheme. It is one of the few enzymes that is able to cleave thioether bonds without using a redox cofactor [22]. Numerous studies indicate that LuxS is highly conserved among several species, including *A. hydrophila, E. coli, V.cholera,* and *S. typhi,* but does not share homology with other functional genes [23,24]. In addition, a number of LuxS protein structural studies have been published. The crystal structure of LuxS indicated that it is homodimeric, with six alpha-helices that are covered with eight stranded beta-barrels. Further, the active site coordinates a zinc ion that binds with several highly conserved residues, including His-54, His-58, and Cys-126 [25,26]. Furthermore, several studies have demonstrated that conformational changes in the protein results in the participation of the 125–131 residues in the restriction active site, and which are closely located to the N-terminus, [26]. Several crystal structures of LuxS have been solved from various bacterial species (*Deinococcus radiodurans*, *Helicobacter pylori*, *Haemophilus influenzae*, *Bacillus subtilis,* and streptococcus mutans) [25,26,27]. *A. hydrophiala* LuxS share sequence similarity 42, 38, 37, 37 and 79% to LuxS from *D. radiodurans*, *H. pylori*, *H. influenzae*, *B. subtilis* and *S.mutans*, respectively [28].

Previous studies have indicated that LuxS has evolved since the divergence of prokaryotic phyla, and that its evolutionary history is congruent with the ribosomal RNA tree of microorganisms. Genome-wide studies of *luxS* have indicated that LuxS is commonly found within the bacterial domain. Concomitantly, the AI-2 mediated signaling process functions as a universal mode of interspecies communication [29]. Accordingly, numerous reports have shown that AI-2 binds receptors, and is a mediator of QS in several species, including those of the *Vibrionales* order, and pathogenic gut bacteria [30].

Large-scale bacterial genome sequencing projects have led to increased recognition of the role of LuxS in the growth and virulence of various bacterial pathogens. Consequently, it is necessary to identify the structure and function of the LuxS protein in the important pathogen, *A. hydrophila*. Herein, the 3D structural model of the *A. hydrophila* LuxS was aligned with a known homologous structure via homology modeling and the threading method (i.e., fold recognition method). Furthermore, predictive computational approaches were used, including sequence analyses, model building, structural analyses, and functional annotation. The overall aim of this study is to predict the structure of *A. hydrophila* LuxS and characterize its structure using bioinformatics methods. Moreover, for the validation of the predicted LuxS model, we docked the AI-2 QS inhibitor with the LuxS model and its homologous proteins via the Docking server and experimentally confirmed its effect over AI-2 inhibition. Our results indicate that AI-2 inhibition was decreased and Luxs downregulated at the protein expressional level when an inhibitor molecule was used. In addition, incubation of cultures with the AI-2 inhibitor and OXY antibiotics indicated a much-decreased growth rate of *A. hydrophila* and maybe a possible cocktail treatment for pathogenic infections. 

## 2. Results

### 2.1. Synthesis of AI-2 Using a LuxS Ssystem

**Figure 1:**
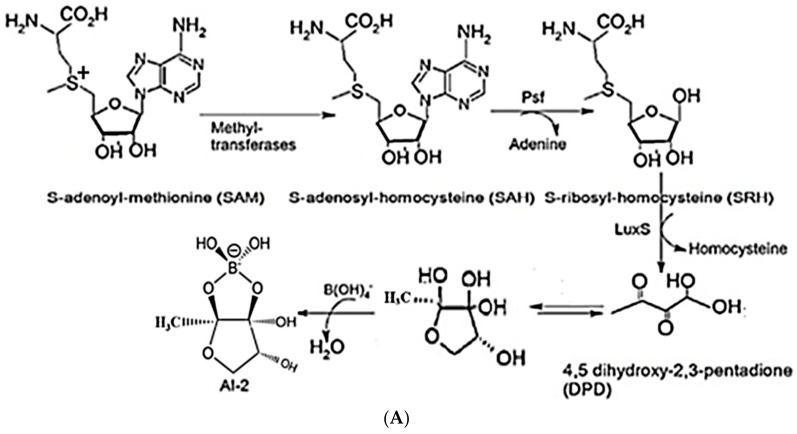

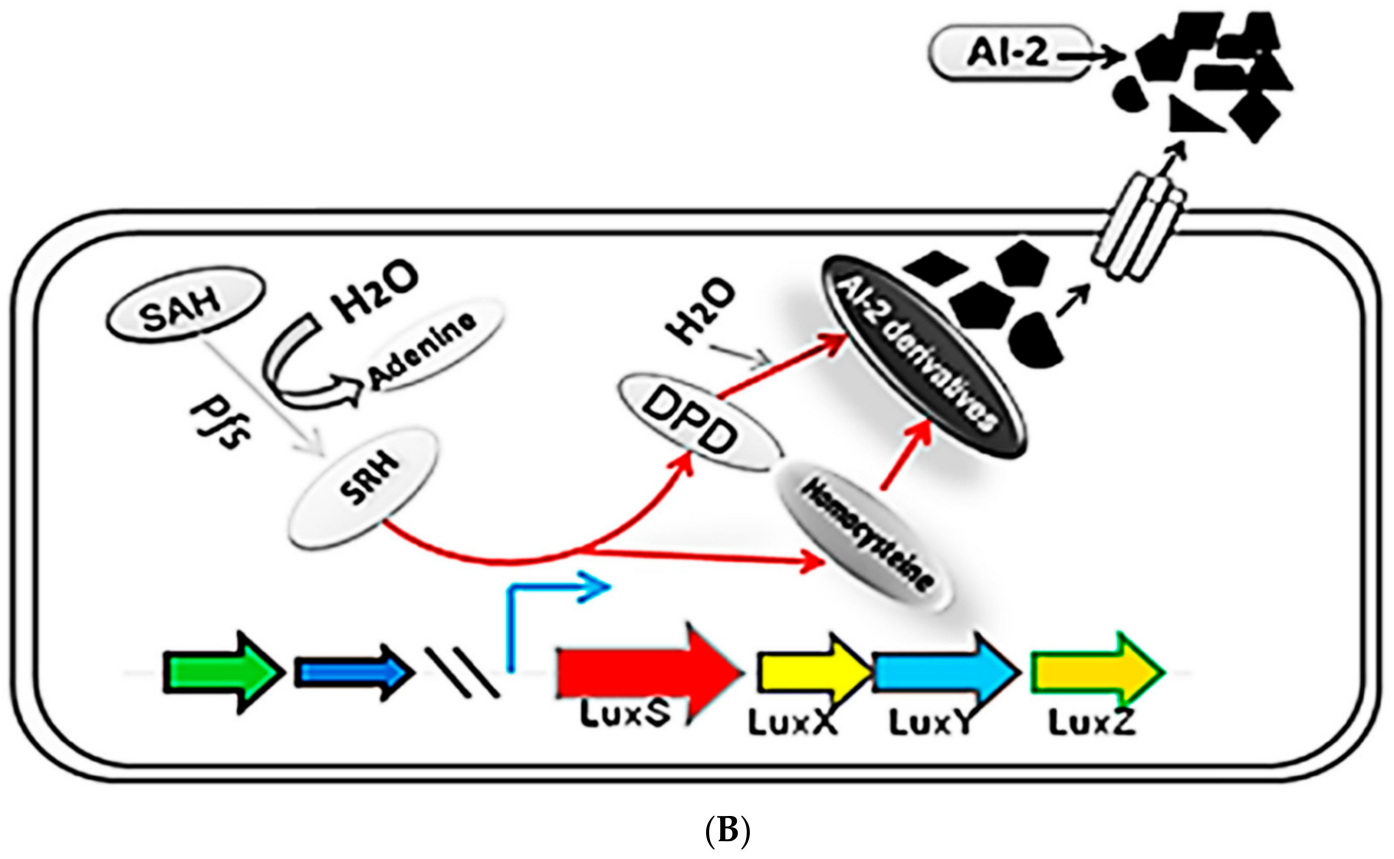
Chemical (**A**) and graphical (**B**) pathways of AI-2 biosynthesis, showing the synthesis of AI-2 via the QS LuxS system in *A. hydrophila*. *luxS* homologs are labeled as LuxX, LuxY, and LuxZ. Pfs converts S-adenosylhomocysteine (SAH) into S-ribosyl homocysteine (SRH) and adenine. LuxS converts SRH into AI-derivatives/precursors such as 4,5-dihydroxy-2,3-pentanedione (DPD) and homocysteine, and DPD spontaneously undergoes cyclization to produce AI-2. AI-2 molecules are emitted from bacterial cells via membrane protein channels, wherein they become active in quorum sensing.

Most bacterial species, including *A. hydrophila*, have conserved *luxS* homologs that are responsible for the production of LuxS enzymes. The conversion of S-adenosylhomocysteine (SAH) by detoxification with the aid of 5′-methylthioadenosine/S-adenosylhomocysteine nucleosidase (i.e., the Pfs enzyme) results in the S-ribosyl homocysteine (SRH) substrate and the removal of an adenine group. Only the LuxS enzyme has been predicted to cleave the substrate (SRH) into 4,5-dihydroxy-2,3-pentanedione (DPD), which is the precursor of AI-2, and homocysteine [14,31]. DPD then spontaneously undergoes cyclization into AI-2 [32,33] (Figure 1A,B). Most bacterial species use Pfs and LuxS to produce DPD, which is then responsible for the biosynthesis of AI-2.

### 2.2. LuxS Sequence Analysis

**Figure 2:**
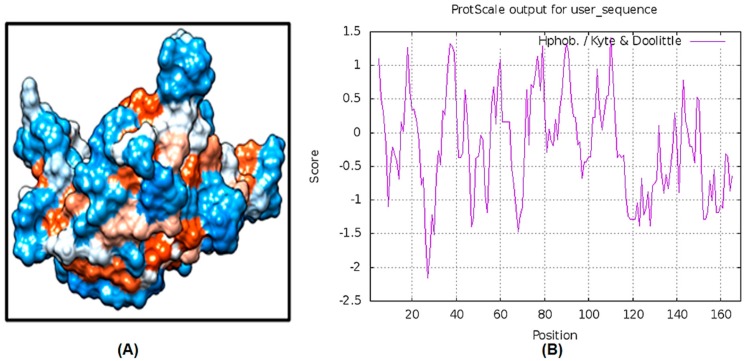
Analysis of LuxS protein hydrophobicity. (**A**) the hydrophilic areas of the LuxS protein are shown in blue, and hydrophobic regions are shown in red, while white represents areas with hydrophobicity values of 0.0. (**B**) Kyte and Doolittle hydropathy plot showing that the LuxS protein is moderately hydrophilic.

LuxS sequence analysis indicated a protein length of 169 amino acids, a molecular weight of 18.79 kDa, and 2618 total atoms. The net charge and isoelectric point of the protein are provided in Supplementary Table S1. The grand average hydrophobicity (GRAVY) index was 0.179, indicating that the protein is hydrophilic (Figure 2A). Kyte and Doolittle hydropathy plots further confirmed the hydrophobicity of the protein (Figure 2B). The hydrophobicity of protein helps to decrease its surface area and reduces unfavorable interactions with water due to keeping it biologically stable and active.

### 2.3. Cellular Localization and Antigenic Site Prediction

**Figure 3:**
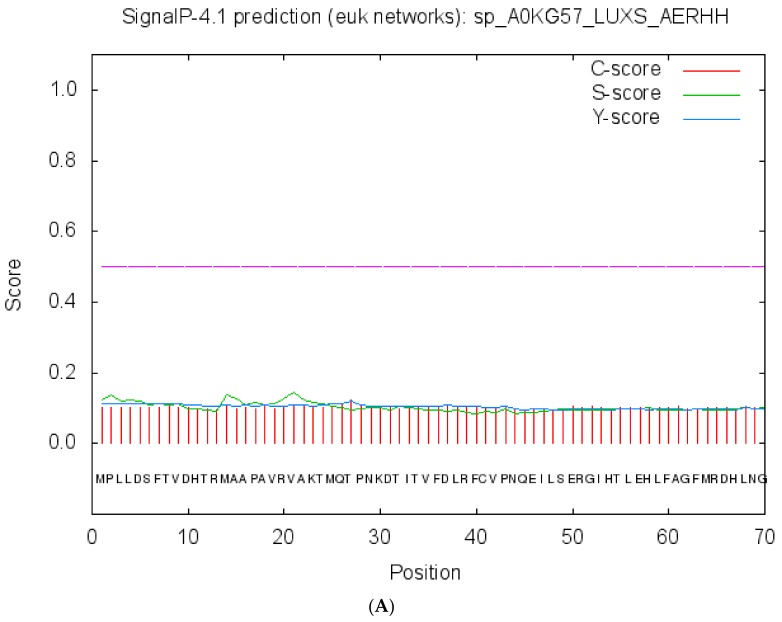

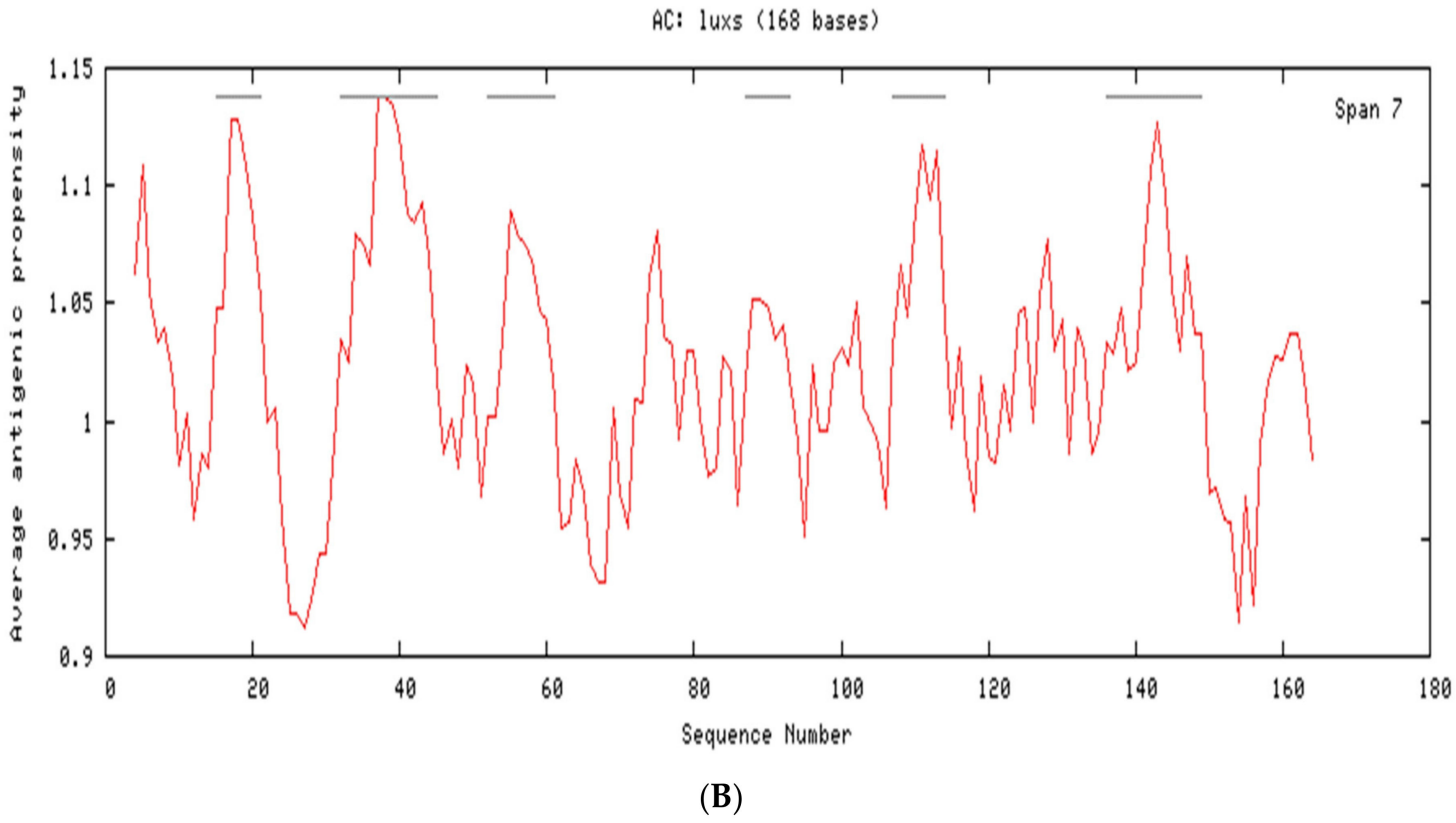
Signal peptides and antigenic determinants. (**A**) prediction of LuxS signal peptides by the Signal peptide server. Signal cleavage sites, internal helices, and associated motifs were not present in the sequence. (**B**) antigenicity profile and antigenic determinants of LuxS. Grey lines show the positions of six antigenic determinants within the LuxS protein.

Protein functions are mostly confined to specific locations. Hence, prediction of protein localization allows for understanding of specific pathways and mechanisms, including the QS pathway or other disease-relevant pathways. Localization analysis using the Predict Protein and CELLO v2.5 servers indicated that LuxS is a cytoplasmic protein, and the highest predicted cellular localization score value was 4.504 (Supplementary Table S1). Signal peptides and internal helices or motifs were not identified inside of the LuxS protein (Figure 3A), which has not been previously reported. Previous analysis shows the presence of hydrophobic residues (valine, cysteine and leucine) on the surface of the protein, which is likely to be the part of antigenic determinants. Analysis with the predicted antigenic peptides tool indicated that LuxS contains six antigenic determinants, with an average antigenic propensity of 1.0223 (Figure 3B). These six antigenic determinants consist of sequence fragments that start and end at different positions: 15–21: AAPAVRV, 32–45: TITVFDLRFCVPNQ, 52–61: GIHTLEHLFA, 87–93: FYMSLIG, 107–114: AMSDVLTV, and 136–149: LEEAHAIARHVLER (Table 1). These predicted antigenic sequence segments may function as stimulators of antibody responses.

Sequence analysis of LuxS indicates that it contains a single metalloenzyme LuxS/M16 peptidase-like domain comprising amino acids in positions 4–162. Metalloenzyme domains exhibit two-layer alpha/beta structures, as in LuxS (S-ribosyl homocysteine; EC: 4.4.1.21), while metallopeptidases share high similarity with the M16 family. These domains share identical active site motifs of HxxEH within the core helix, but differ in one of the metal-binding residues. In contrast, LuxS is an iron-dependent homodimer metalloenzyme that holds two tetrahedral metal-binding sites that are identical to peptidases and amidases, and also has an extra N-terminal strand [26,34] (Supplementary Figure S1).

### 2.4. LuxS Structural Analysis

**Figure 4:**
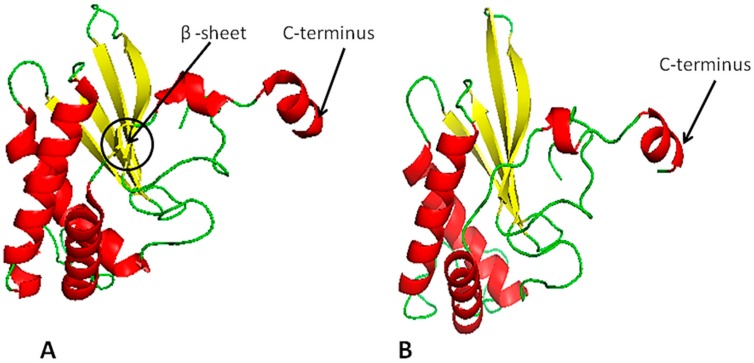
(**A**) LuxS homology model and (**B**) predicted 3D-structural model of the LuxS protein. Red indicates alpha helices, yellow indicates sheets, and green indicates loops. The difference between template and predicted model is found. The template contains one extra beta sheet shown within the black circle (about two residues long) and has long alpha helices. The predicted model at C-terminus alpha helix has short, long loops and one residue found is the loop at C-terminus, while the template does not contain any loop residue at the C-terminus.

The homology modeling of LuxS was performed based on known crystal structures of *Salmonella typhi* (PDB ID: 56e8), *Deinococcus radiodurans*, (PDB ID: 1VJE) and *Streptococcus suis* (PDB ID: 4XCH) and the template (PDB ID: 56e8) was selected with highest 78% structural similarity (Figure 4A). Furthermore, to better understanding the details of LuxS structure in *A. hydrophila* and its differences with template, the structure prediction indicates that LuxS is comprised of 31.36% loops, 41.42% helices, and 17.16% strands. Further differences between model and template are shown in (Figure 4A,B). The tertiary structure of LuxS was accurately predicted using threading methods in the i-TASSER and Phyre2 servers [35,36,37]. Quality analysis of the predicted model was conducted using several SAVES (Procheck, Errat, Prove and Verify-3D) servers [38,39,40]. The QMEAN4 server was used to validate the results, evaluate the Psi/Phi values of the Ramachandran plot, and other quality-filtration analyses against non-redundant sets of proteins from data banks. These analyses indicated that the i-TASSER’s predicted 3D model (Figure 4B) exhibited higher accuracy than the Phyre2 predicted 3D-model.

The model was selected based on the i-TASSER results and higher QMEAN4 scores. The SWISS-PDB viewer was used to model energy minimization and refine the model. The LuxS ‘Model1’ with a QMEAN4 value of −3.17 and z-score value of −6.53 was selected for energy minimization. PROCHECK was then used to analyze the structural and stereochemical properties of the protein by recognizing overall and residue-by-residue geometry. A Psi/Phi Ramachandran plot was used to assess the quality of the model, and indicated that 86.2% of the residues were observed in the most favored regions, 8.4% were within additionally allowed regions, and 5.4% were in outlier regions, while none of the residues were observed in disallowed regions (Supplemental Information S1). The reliability of the model was assessed via ERRAT, which examines the statistics of non-bonded interfaces between diverse atom types. The value of error functions versus positions in a nine-residue sliding window was calculated by comparing statistics against highly refined structures [41]. Quality assessment via ERRAT and PROVE indicated that the model statistics were appropriate, consequently validating the build models (Supplemental Information S2 and S3). Thus, model validation suggested that the model adequately represented the native protein. 

### 2.5. Functional Annotation of LuxS

Functional annotations of the hypothetically predicted proteins were conducted using the Predict and ProFunc servers. These analyses suggested that the protein was involved in various biological and biochemical functions. Specifically, LuxS is involved in quorum sensing, biosynthetic processes, catalytic activities, metal ion binding, cation binding processes and lyase activity, and S-ribosyl homoserine lyase activities. Moreover, LuxS was also predicted to have iron ion binding activities.

### 2.6. Natural Ligand, Its Binding Sites and Analysis of Topological Features

**Figure 5:**
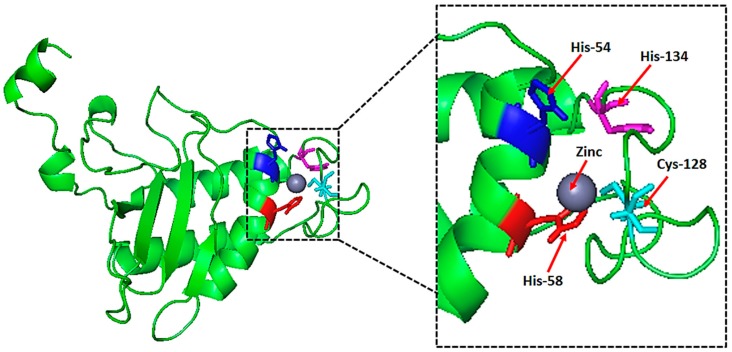
Predicted natural ligand and its binding sites in the predicted model of LuxS. A ZINC-ligand (sphere) was predicted using the COACH sever, as based on high MAMMOTH scores. Binding sites are designated with amino acid numbers.

A zinc (Zn) ion ligand was predicted based on maximum MAMMOTH scores values. In particular, the Zn ion binds via the four amino acids HIS-54, HIS-58, CYS-128, and HIS-134 (Figure 5). Furthermore, the same Zn ligand was predicted to bind to HIS-54, HIS-58, and CYS-128 using the COACH-meta server, as determined with high scores (Figure 5).

The active site center of LuxS is formed by conserved residues, as determined via sequence alignment analysis. Nearly 30 residues were identified in the conserved region (Supplementary Figure S2B). The metal cofactor is found in the center of the conserved cluster (Supplementary Figure S2A), which is consistent with the identification of its active site. The initial identification of the metal cofactor was predicted using the COACH-meta server, as described above. The HIS-54, HIS-58, and CYS-128 residues are coordinated with the metal ligand by single monomer chains. HIS-54 and HIS-58 are found in the central region of α1, and harmonize with the metal via N-terminal atoms. In contrast, CYS-128 is present in the extended loop. These residues and water molecules complete the formation of the coordinated sphere, and zinc binding takes place with a tetrahedral geometry of residues. The relevant interatomic distances were: HIS 58 NE-Zn, 1.902 Å; HIS 54-NE Zn 2.821 Å, CYS 128 Sg-Zn, 2.611 Å; and O (HOH)-Zn, 1.82 Å (Supplementary Figure S2A). The geometry and the presence of the solvent ligand indicate that the metal molecule plays more of a catalytic role rather than a structural one. In addition, the above-mentioned sequences and motifs are similar to those of metal binding sites and conserved sequences also observed in peptidases and amidases. In contrast to those enzymes, LuxS may function in the biosynthesis of autoinducer-2 (AI-2) by acting as a hydrolase [26]. Regardless, these data provide the framework for experimental analysis of interatomic molecular interfaces in order to investigate the production of AI-2, and its effects on quorum sensing.

### 2.7. Virtual Screening and Toxicity Studies

The structure-based ligand search was done using a DOCK blaster against drug like subset molecules of the ZINC database. The server predicted a possible ligand binding sites in the structure of the protein. The ligand binding sites were chosen for the HTS against subset of molecules. Based on ligand binding, descending affinity with protein 180 HTS was generated. Furthermore, for the subsequent study, the top nine ligands were selected. 

The OSIRIS property calculator used to study the toxic effect of the selected seven-ligands molecules. Out of the seven compounds, six were found to be non-toxic (Table 2). The compound ‘3-(1,2-diazabicyclo[2.2.2]oct-2-yl)-1-phenylpropyl acetate’ had its toxicity as a mild irritant and also had medium reproductive effects. Therefore, this molecule was removed for docking. The remaining six molecules were screened on the basis of a Clogp value, solubility, molecular weight, drug-likeness and the drug score. For further docking analysis, a molecule with the highest drug score was selected. (Table 3).

### 2.8. Molecular Docking

**Figure 6:**
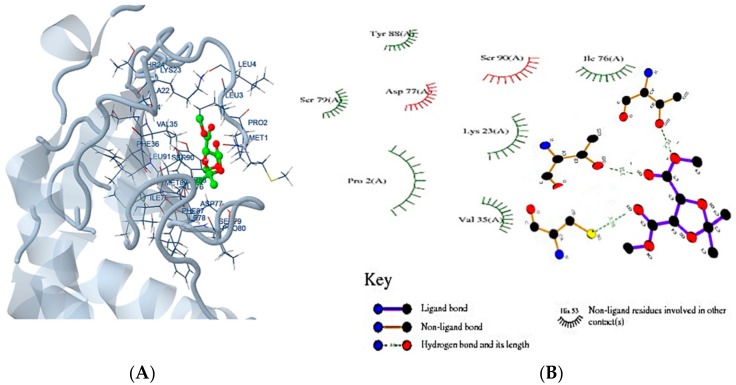
(**A**) ligand-protein complex docked by dock server (**B**) the schematic illustration interaction of dimethyl (−)-2,3-*O*-isopropylidene-l-tartrate ligand molecule with predicted LuxS protein model.

Docking Server is a protein-ligand docking online program that uses the Lamarckian genetic algorithm (LGA) programming. The docking of selected ligand (dimethyl (−)-2,3-*O*-isopropylidene-l-tartrate) was performed with all homologous proteins. The docked protein-ligand complexes were analysed based on the best binding affinity. It was found that a LuxS predicted model of had a best binding affinity (−3.06 Kcal/mol) with dimethyl (−)-2,3-*O*-isopropylidene-l-tartrate’ (Table 4; Figure 6A,B), and the docking of homolog proteins with ligand is shown (Supplementary Figures S3–S5).

### 2.9. LuxS AI-2 Biosynthesis Inhibition Using a (−)-Dimethyl 2,3-O-isopropylidene-l-tartrate Inhibitory Compound

**Figure 7:**
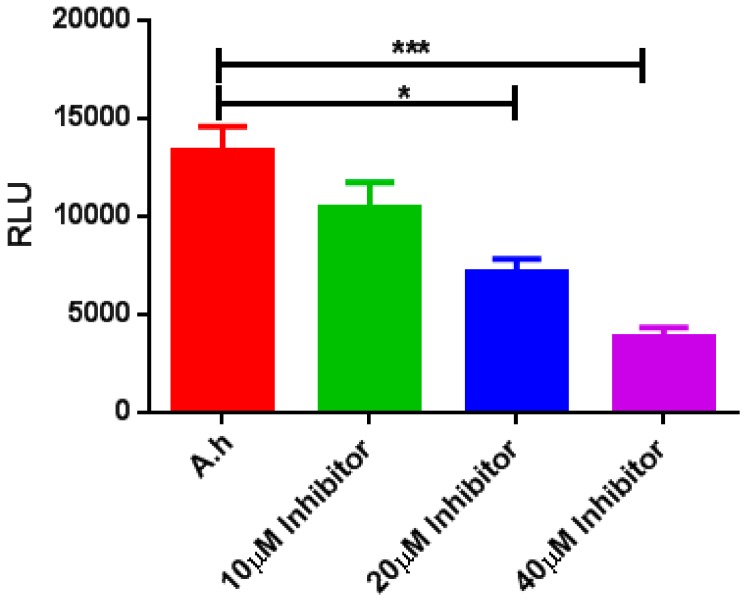
Detection of AI-2 activity via bioluminescence assay of *V. harveyi* BB170 incubated with culture supernatant of *A. hydrophila* in the absence (control) and presence of the inhibitor (−)-Dimethyl 2,3-*O*-isopropylidene-l-tartrate. The bioluminescence measurement was performed seven hours after the addition of the inhibitor. Bioluminescence was lower than that of the untreated control (*p <* 0.001 *** and *p <* 0.05 *). The error bars were calculated using a standard error of mean (SEM). *A.h* (*A. hydrophila* wild type).

The effect of LuxS AI-2 QS inhibition was assessed using bioluminescence assays of *V. harveyi* BB170 and *A. hydrophila* supernatant in order to determine LuxS AI-2 activity. The (−)-Dimethyl 2,3-*O*-isopropylidene-l-tartrate inhibitory compound was applied at various concentrations, and the consequent inhibitory effects were observed (Figure 7). AI-2 inhibition with the inhibitor at a 40 μM concentration was significantly reduced compared to the control, while the 10 and 20 μM treatments also exhibited correspondingly lowered activities of LuxS and this experiment was repeated three times.

### 2.10. Validation of AI-2 Inhibition via Analysis of Protein Expression Levels

**Figure 8:**
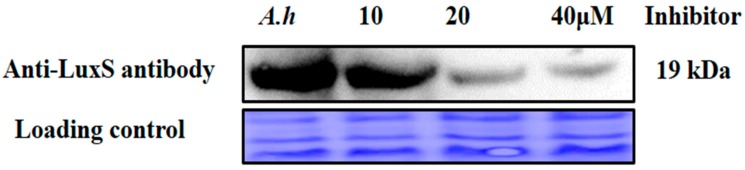

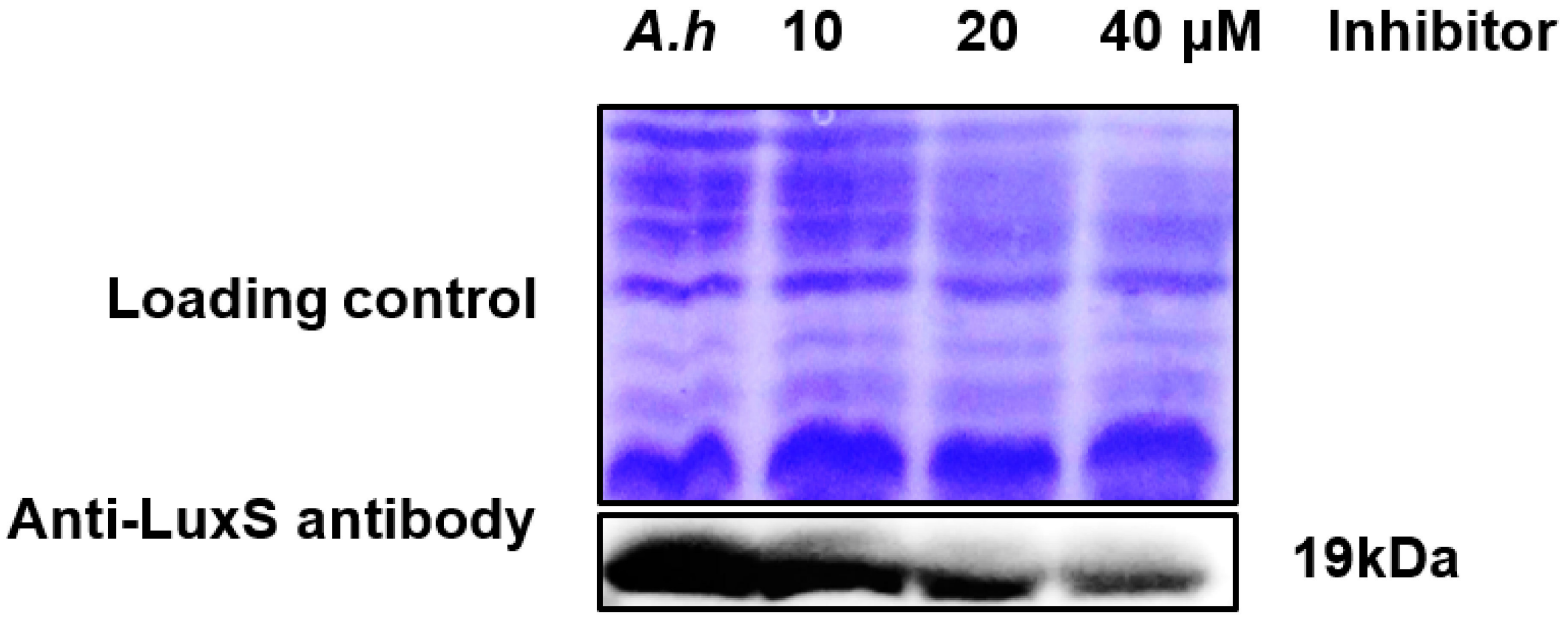
Validation of LuxS protein expression levels with Western blotting using increasing concentrations of a LuxS inhibitor compound. *A. hydrophila* was treated and untreated (*A.h*, wild type used as a control) with increasing inhibitor concentrations of 10, 20, and 40 μM (lower panel). Coomassie R-350 staining of the membrane shows equal loading of the protein sample (upper panel).

Further validation of AI-2 inhibition was conducted at the protein expression level via Western blotting with the anti-LuxS antibody. The sample treated with the (−)-Dimethyl 2,3-*O*-isopropylidene-l-tartrate inhibitor compound at 40 μM exhibited downregulation of LuxS protein expression compared to expression with inhibitor at 10 and 20 μM concentrations (Figure 8). These results are consistent with the observed AI-2 biosynthesis inhibition, further validating our biochemical model.

### 2.11. The Mixture of OXY and AI-2 Inhibitor Is a Potentially Synergistic Strategy for Bacteriostasis

**Figure 9:**
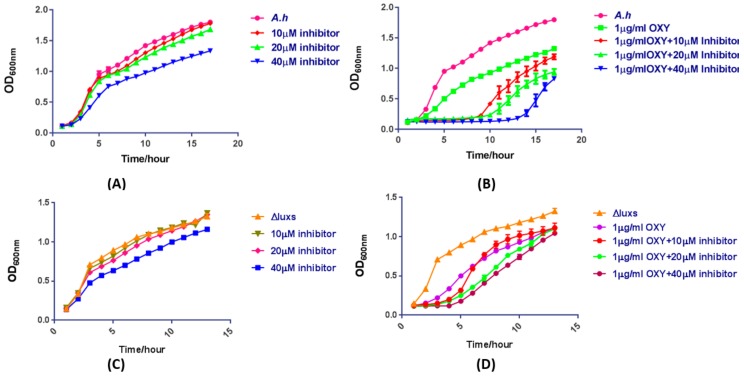
Effects of incubation with the (−)-Dimethyl 2,3-*O*-isopropylidene-l-tartrate LuxS inhibitor compound on growth of *A. hydrophila*. (**A**) growth of *A. hydrophila* when treated without or with (−)-Dimethyl 2,3-*O*-isopropylidene-l-tartrate inhibitor compounds at increasing concentrations; (**B**) the effect of cocktail therapy on *A.hyrophila* growth, when treated with increasing concentrations of the LuxS inhibitor and 1 μg/mL OXY (**C**,**D**) *luxs* knocked out (Δ*luxS*) strain treated versus untreated with inhibitor plus cocktail therapy with 1 μg/mL OXY respectively are shown here. The error bars were calculated using standard error of mean (SEM).

To further validate the effect of AI-2 inhibitor on AI-2 biosynthesis and *bacteriostasis* when synergisted with antibiotics, (−)-Dimethyl 2,3-*O*-isopropylidene-l-tartrate was used to assess differential responses in *A. hydrophila* strain under AI-2 biosynthesis inhibition. Cell growth was first measured (as an optical density (OD) values) and were compared between treated and untreated strains incubated with various concentrations of the inhibitory compound. Incubation with 40 μM inhibitor resulted in clearly lowered growth compared to incubations with either 10 or 20 μM of the inhibitory compound (Figure 9A). The growth curve of *A. hydrophila* presented not different when treated with low dose concentration OXY antibiotics alone. We then evaluated the survival rate of *A. hydrophila* strain when exposed to 1 μg/mL OXY and the inhibitory compound. A very slow growth rate was observed in the treated strains compared to the untreated strains. Incubation with the AI-2 inhibitor and 1 μg/mL OXY resulted in a much lower survival rate at a 40 μM concentration of inhibitor compared to that of the incubations with 10 and 20 μM inhibitor (Figure 9B). The results clearly showed that the mixture of OXY and AI-2 inhibitor should be a potentially synergistic strategy for *bacteriostasis* in *A. hydrophila*. For further confirmation, we have performed experiments by using a knocked out Δ*luxS* strain. Results showed that there is not a significant difference among *A. hydrophila* wild-type, ∆*luxS* treated with serials of inhibitor ((−)-Dimethyl 2,3-*O*-isopropylidene-l-tartrate) concentrations alone although the growth curve with 40 μM treatment showed slightly decreased. Moreover, the growth rate of *A. hydrophila* treated with 1μg/mL OXY antibiotics plus inhibitors showed a more significant decrease than ∆*luxS*. These results showed their consistency with docking results, as the *luxS* mutant strain has a significant growth difference while compared with the *luxS* coding strain of *A. hydrophila* (Figure 9C,D).

## 3. Discussion

*A. hydrophila* causes a fulminant epidemic in aquatic organisms, including other severe bacterial diseases, like hemolytic ascetic disease, fulminant hemorrhagic and bacterial septicemia [42]. AI-2 synthesis has been thoroughly documented in various bacterial species over the last several decades, although it was originally described in *V. harveyi*. AI-2 functions as a general communication signal in bacteria and several studies have demonstrated that Gram-negative and Gram-positive bacteria sense and respond to AI-2. Thus, the enzyme responsible for producing AI-2, LuxS, is an essential contributor to quorum sensing for inter-species communication at high cell densities [7,13,43]. AI-2 can also provide information on additional cellular physiological information more than other autoinducers, because its biosynthesis is directly linked to cell growth, and consequently provides information on the fitness of bacterial populations [7]. In the aquaculture industry, conventional strategies were used for the inhibition of QS. Several antimicrobial compounds have been applied to kill or inhibit the bacterial by means of interfering with important activities like DNA synthesis, membranes, and other proteins. By the rapid use of antibiotics for the control of bacterial disease aquaculture has posed threat to public health as well as antibiotics resistance. It very essential for aquaculture industry to develop novel antibacterial drugs to face the emerging resistant strains for the control of the bacterial disease. QS inhibition is a possible strategy to combat resistant strains [44]. Therefore, various natural products, enzymes, and chemicals interfere with QS have been identified. The luxS-based AI-2 QS system was found in *A. hydrophila* its mutant showed the alteration in biofilm synthesis, increased virulence observed in a mouse model, and a decrease in motility [45]. Alfaro et al. has synthesized two substrate analogs of intermediates of the first and last steps in AI-2 synthesis and QS inhibition has observed [46]. In *P. aeruginosa* AHL synthesis was decreased using sodium houttuyfonate [47], and it also indicated sodium houttuyfonate inhibits QS in *A. hydrophila*. Halogenated furanones disturbs the non-AHL-based QS, including AI-2 and Gram-positive systems [48]. Untill today, a few studies have been pointed out the possibility of the potential resistance to QS inhibitors and in some situations, quorum sensing inhibition has been considered it does not kill bacteria [49,50]. After that, at the time of administration, these inhibitory compounds’ dose have increased, which inhibits growth [51,52]. However, quorum sensing inhibition has a significant strategy to control fish disease. Here, we investigated the biosynthesis of the AI-2 QS-associated LuxS protein using in silico methods, followed by experimental validation of the predicted model via using (−)-Dimethyl 2,3-*O*-isopropylidene-l-tartrate inhibitor. Our results indicate that AI-2 is a potential target for the inhibition of bacterial communication [53].

A detailed understanding of the physicochemical properties of the LuxS protein, including its quaternary structure, antigenicity, and structural and functional properties, would be helpful for identifying its role in quorum sensing. In silico modeling of several unknown protein structures using bioinformatics tools has been particularly useful in inferring these properties. Little information was previously available for the crystal structure of the LuxS protein. Consequently, we predicted its structure using in silico methods to aid in its exploration and identify drug targets and diagnostic markers for hosts infected with the aquatic pathogen *A. hydrophila*. *A. hydrophila* LuxS possesses essential communication characteristics but is unique compared to other S-ribosyl homocysteine lyases due to its metal binding, ion binding, and quorum sensing activity [54]. LuxS is predicted to be a cytoplasmic protein, wherein it can readily produce AI-2. The protein contains six antigenic determinants and a metalloenzyme domain, suggesting the presence of important metal binding sites, similar to peptidases and amidases [55,56]. The LuxS cytoplasmic protein exhibited low QMEAN4 score and Z-score values for the reconstructed structure. Furthermore, structure validation was confirmed by Psi/Phi Ramachandran plots. Hence, the model appears to be of high quality, and suitable alternative drug targets may be identified based on the protein structure reported herein. The successful prediction and validation of the LuxS structure indicated greater than 95% accuracy, suggesting a high likelihood that the predicted structure is accurate compared to the native protein. Accurate torsion angle conventions were observed in the structure and improper dihydral angles that normally are present, were also observed. No atoms were missing from the structure and improper dihydral angles RMS-scores were within normal ranges. Further, all of the necessary oxygen atoms were present at the C-terminus.

QS is an essential regulator of bacterial virulence, and its inhibition has high potential as an alternative strategy for the treatment of bacterial infections. *luxS* is responsible for the production of AI-2 QS signal molecules, commonly referred to as AI-2 molecules [57]. As previous studies revealed that the *luxS* isogenic mutant of *A. hydrophila* altered biofilm formation dynamics and its architecture, bacterial motility reduced and virulence has been enhanced observed in a mouse model [58] and *luxS* mutant strain failed to produce AI-2 as studied in *Aeromonas salmonicida* [59] Another report shows that tannic acid is also a potential QS inhibitor, which reduces biofilm formation, swarming motility and blood haemolysis activity of fish pathogenic bacteria [60,61]. Moreover, 5906 a small peptide binds with Luxs and prevents the synthesis of functional LuxS homodimer of *Edwersilla tarada* [62]. For the study of AI-2 inhibition of *A. hydrophila*, a suitable inhibitor was found by HTS against a drug-like subset Zinc database via an online Dock blaster program and finally docked with a LuxS predicted protein model and its homologous proteins using a docking server online server [63]. The 180 hits were generated based on ligand binding descending affinity with a target protein and the top seven molecules have been selected for toxic studies. From toxic studies, six molecules were found to be non-toxic and were screened further for docking analysis. Based on the best drug score (0.97), (−)-Dimethyl 2,3-*O*-isopropylidene-l-tartrate molecule was selected for docking purposes. Molecular docking studies show that this inhibitor has a very best binding affinity with the LuxS predicted model (−3.06kcal/mol) as compared to its homologous proteins (Table 4). Therefore, the results reported here confirm the QS inhibitory activity of the AI-2 inhibitor (−)-Dimethyl 2,3-*O*-isopropylidene-l-tartrate. AI-2 bioluminescence synthesis was inhibited, and our data indicate that it exerts an inhibitory effect on the production of AI-2. Moreover, AI-2 inhibition was confirmed at the protein expression level, with down regulation of LuxS expression, thereby inhibiting AI-2 QS signaling molecule production when cells were incubated with the inhibitor at a 40-μM concentration. The LuxS did show lower expression in the treatment of inhibitor in this study. The real reason of this phenomenon is still unknown, however, it is possible this small molecule does not only bind with LuxS but may bind to other proteins and has off-target effect, resulting in downregulation of LuxS expression level. As shown in previous studies, several DNA-binding regulatory proteins are allosteric in nature, change their activities at the time of binding to small metabolic intermediates or inhibitors or ligands and responds to physiological changes via the pattern of gene expression. For example, a known *E. coli* tryptophan aporepressor (TrpR) binds to the operator region and represses the expression of *trp* gene. While tryptophan exogenously provided to Δ*trp* mutants has shown overexpression and is the positive regulator [64,65]. The lower expression of LuxS may trigger a negative regulator during (−)-Dimethyl 2,3-*O*-isopropylidene-l-tartrate treatment in this study. Finally, the growth and survival of *A. hydrophila* was analyzed with various concentrations of the AI-2 inhibitor, and in the presence of OXY. AI-2 biosynthesis decreased considerably upon treatment with the cocktail, including 1 μg/mL OXY. There are many of researchers that havedocumented the effect of LuxS on bacterial antibiotics resistance, which may be in part because LuxS relates to quorum sensing and biofilm formation and finally affect bacterial antibiotics resistance [66,67]. Besides this, it was reported that LuxS was also involved in metabolic pathways such as activeated methyl cycyle and sulfur metabolism and may finally lead to the fluctuation of intracellular metabolic flows. Recently, Peng et al. reported that antibiotics resistance relates to several metabolic pathways and exogenous metabolites plus antibiotics would be boosted to kill antibiotics resistant bacteria [68]. Thus, LuxS may affect bacterial multi-drugs resistance via complicated regulation mechanisms. In this study, we used oxytetracycline whose resistance was reported to be affected by LuxS, to combine with inhibitor (−)-dimethyl (−)-2,3-*O*-isopropylidene-l-tartrate and to value its bacteria-killing activity [69]. Thus, a cocktail of OXY and the inhibitor may be an efficient strategy for antimicrobial therapy and blocking AI-2 biosynthesis. These results are consistent with previous studies indicating that cocktail analogous therapy efficiently decreases AI-2 biosynthesis in *P. aeruginosa* [70].

The results described here provide a foundation for understanding the structure and function of the LuxS protein that is critically involved in the quorum sensing. The use of numerous computational and bioinformatics tools has proven useful in the reduction of costly experiments for drug or vaccine discovery. Comprehensive analysis of the post-translational modification of the protein is required in order to gain insight into the conformational changes of proteins, and is thus a potential target for future studies. Lastly, protein protein interactions, protein-ligand interactions, and binding efficacy of co-factors via docking studies are made possible by structural modeling analyses, as are described here for LuxS. These analyses provide a framework for identifying novel drug molecules that can aid in the treatment of diseases caused by *A. hydrophila*.

## 4. Methodology

### 4.1. Bioinformatics Analyses

#### 4.1.1. Retrieval of Protein Sequences and Analysis

The LuxS amino acid sequence was retrieved from the Uniprot database (A0kG57) and the ClustalW aligner was used to align the sequence and assess its appropriateness for protein structure prediction. The LuxS protein contained the intact sequence of amino acids comprising the N-terminal and C-terminal ends, and was thus suitable for structural prediction. Structurally homologous sequences were retrieved from the protein data bank (PDB) after comparison using a sequence similarity model.

#### 4.1.2. Assessing Physicochemical Properties

The target LuxS protein sequence was used as the template in determining its molecular profile using the Portparam tool of ExPASy. In addition, the predict protein server was used to determine the solubility of the protein. The SOPMA, SAPS, and FindMod software packages were used to analyze the structural properties of the protein. Subcellular localization predictions were performed using PSortB and CELLOv2.5. The presence of signal peptides within the amino acid sequence was determined using the SignalP 4.1 server. Lastly, the Antigenic Peptides program was used to predict the antigenicity of the protein.

#### 4.1.3. Structural Modeling

Similarity of the LuxS protein with other publically available homologs was assessed by searching against non-redundant databases including NCBI and PDB. The amino acid similarity between the target (query) and the template was observed as 78%. Prediction of structural folds was conducted with structure fold recognition techniques, as implemented in the i-TASSER and Phyre2 perdition servers. The presence of additional functional domains was determined using the InterPro protein family database and the Evolutionary classification of protein domains (ECOD) database.

#### 4.1.4. Structure Validation and Refinement

The protein structure was generated using the i-TASSER and Phyre2 servers and validation was conducted using the SAVes server. The QMEAN6 program within the SWISS-MODEL workspace was then used to determine the quality of the structure. The energy levels were minimized, and the structure was improved based on a Ramachandran plot. Lastly, the modeled structure was visualized using the PyMOL v1.7.4.5 program.

#### 4.1.5. Active Site, Ligand, and Ligand Binding Sites Evaluation

The computed atlas of surface topography of proteins (CASTp) server was used to identify the active sites. The server locates and measures concave surface regions of modeled proteins. In addition, the 3D ligand binding site prediction server was used to investigate ligand characteristics and potential binding sites. Further binding site verification was conducted using the COACH server.

#### 4.1.6. High Throughput Virtual Screening and Toxicity Analysis

High throughput (HTS) was done by using of DOCK blaster [71], which is a structure based ligand discovery online server of University of California, San Francisco, CA, USA. The protein and the active sites were submitted to the server and suitable ligand binding sites shown by DOCK blaster. That active site of the protein was docked against the drug like subset of the ZINC database.

The toxicity of the ligand molecules was analysed via OSIRIS property calculator. This online server reveals the several properties of ligand molecules such as Mutagenic, Tumorigenic, and Irritant, Reproductive effective, Clogp value, Solubility, Molecular weight, Drug-likeness and finally the Drug score.

#### 4.1.7. Ligand Preparation and Molecular Docking of the Receptors to Ligands

The (−)-Dimethyl 2,3-*O*-isopropylidene-l-tartrate inhibitor was selected based on the best drug score for further study. The ligand was retrieved in smiles file (ZINC: 00056779) as (−)-Dimethyl 2,3-*O*-isopropylidene-l-tartrate. Then, docking calculations were carried out using Docking Server [72]. Gasteiger partial charges were added to the ligand atoms. Non-polar hydrogen atoms were merged, and rotatable bonds were defined. Docking calculations were carried out on zinc_56779 protein model. Essential hydrogen atoms, Kollman united atom type charges, and solvation parameters were added with the aid of Auto Dock tools [73]. Affinity grid (Box size: 20 × 20 × 20 Å and box center: 58.88 × 58.99 × 58.64 for x, y, and z, respectively) and 0.375 Å spacing were generated using the Autogrid program [74]. Auto Dock parameter set- and distance-dependent dielectric functions were used in the calculation of the van der Waals and the electrostatic terms, respectively. Docking simulations were performed using the Lamarckian genetic algorithm (LGA) and the Solis and Wets local search method [75]. Initial position, orientation, and torsions of the ligand molecules were set randomly. All rotatable torsions were released during docking. Each docking experiment was derived from 10 different runs that were set to terminate after a maximum of 250,000 energy evaluations. The population size was set to 150. During the search, a translational step of 0.2 Å, and quaternion and torsion steps of 5 were applied.

### 4.2. In Vitro Methods

#### 4.2.1. Bacterial Strains and Growth Conditions

*A. hydrophila* ATCC 7966 was stored in our laboratory at −80 °C and Δ*luxS* strain was constructed using two strains of *E. coli* MC1061 (λpir) and S17-1 (λpir) with suicide vector pRE112 was used to construct mutant by *sacB*suicide gene-based allelic exchange as previously described [1]. The culture was streaked on Luria–Bertani (LB) agar plates, and a single colony was selected the next day and inoculated in 5 mL LB medium for overnight incubation. The culture was then diluted in 100 mL LB at a 1:100 ratio and grown until the OD_600_ reached ~1.0. The *Vibrio harveyi* strain was grown in AB medium for 8–10 h at 30 °C with shaking at 200 rpm. The ligand identified from the ZINC database, (−)-Dimethyl 2,3-*O*-isopropylidene-l-tartrate, was purchased from Sigma-Aldrich (Spruce Street, St. Louis, MO, USA).

#### 4.2.2. AI-2 Inhibition Bioluminescence Assay

*A. hydrophila* was cultured overnight at 30 °C with and without (−)-Dimethyl 2,3-*O*-isopropylidene-l-tartrate at various concentrations: 10, 20, and 40 μM. The culture supernatant was separated using centrifugation at 10,000× *g* for 10 min at 4 °C and then stored at −20 °C. The *V. harveyi* reporter strain was grown in AB media until the optimum OD_600_ was 1.0–1.1, as previously described [76]. *V. harveyi* cultures were diluted 1:5000 in fresh AB medium. Sterile CD medium was then added to the dilution up to 10 mL and 180 μL of the mixture was loaded into the wells of a 96-well plate [43,77]. Finally, 20 μL of the *A. hydrophila* supernatant incubated with and without (−)-Dimethyl 2,3-*O*-isopropylidene-l-tartrate was added, and the stimulation of AI-2 biosynthesis intensity was measured using a SpectraMax^®^i3 Molecular (Molecular Devices, Sunnydale, CA, USA).

#### 4.2.3. Western Blotting

Western blotting was performed as previously described, with slight modifications [78]. Briefly, about 20 μg of *A. hydrophila* ATCC 7966 sample treated with or without 10, 20, and 40 μM (−)-Dimethyl 2,3-*O*-isopropylidene-l-tartrate was resolved on a 12% sodium dodecyl sulfate polyacrylamide gel electrophoresis (SDS-PAGE). The samples were then electrotransferred to a polyvinylidene difluoride (PVDF) membrane using the Trans-Blot Turbo Transfer System (Bio-Rad, Hercules, CA, USA) at 1.3 A for 20 min. They were then blocked with 5% (*w*/*v*) skim milk for 1 h, and the membrane was incubated in a 1:5000 dilution of primary (LuxS) antibody overnight at 4 °C. Following incubation, the membranes were washed five times in phosphate saline buffer (pH 8.0) containing 0.1% (*v*/*v*) Tween (PBS-T). The washed membrane was incubated with horseradish peroxidase-conjugated goat anti-rabbit secondary antibody at a 1:5000 dilution in PBS-T at room temperature for 1.5 h. The immunostained protein was then detected using the Clarity™ Western ECL Substrate (Bio-Rad, Hercules, CA, USA) and scanned with the ChemiDoc MP imaging system using the Image Lab software (Bio-Rad, Hercules, CA, USA).

#### 4.2.4. Antimicrobial Survival after Cocktail Therapy

An antimicrobial survival assay was performed as previously described [1,78]. Wild-type *A. hydrophila*, and *ΔluxS* strains were incubated at 30 °C in 5 mL LB medium overnight. The overnight grown bacterial cultures were then diluted at 1:100 with freshly prepared LB medium in a HONEYCOMB^®^ sterile 100 well plate. The sample was treated with (−)-Dimethyl2,3-*O*-isopropylidene-l-tartrate inhibitor at increasing concentrations of 10, 20, and 40 μM. In addition, diluted concentrations of the antibiotic inhibitor oxytetracycline (OXY) were added at a 1 μg/mL concentration and OD values for growth were measured [79,80]. The OD_600_ were measured for 16 h using a Bioscreen C system (Lab Systems, Helsinki, Finland). Absorbance values were then compared between treated and untreated cultures.

#### 4.2.5. Statistical Analysis

Every experiment was carried out independently three times and values are presented as the standard error of the mean (SEM). The data were evaluated using one way analysis of variance tests. A *p* value < 0.001 was considered to be statistically significant.

## 5. Conclusions

Taken together, LuxS produces AI-2, which is responsible for quorum sensing signaling in the intercommunication of *A. hyhrophila* ATCC7966. In silico threading methods were used to predict the LuxS protein structure, which revealed a similar theoretical structure as other homologous proteins. Furthermore, we predicted and refined the LuxS model and its ligand binding sites/residues LYS 23, VAL 35, PRO 2, and ASP 79 within the LuxS. Additionally, (−)-Dimethyl 2,3-*O*-isopropylidene-l-tartrate inhibitor molecule was identified via HTS and selected based on the best drug score. Then it docked with the LuxS model and its homologous proteins and, finally, the inhibitor was evaluated experimentally by inhibition of AI-2 biosynthesis. AI-2 biosynthesis was inhibited at a 40 μM concentration and the inhibitor shows a potent synergistic effect of bacteriosis and thus substantiated themselves as a therapeutic on *A. hydrophila*. These results provide a framework to further develop a complete understanding of LuxS, and implicate the need for additional structure-level experimental investigations.In addition, this information should assist in the discovery of a high potential antibacterial drug against *A. hydrophila* as well other microbes.

## Figures and Tables

**Table 1:** Antigenic determinants within LuxS.

n	Start Position	Sequence	End Position
1	15	AAPAVRV	21
2	32	TITVFDLRFCVPNQ	45
3	52	GIHTLEHLFA	61
4	87	FYMSLIG	93
5	107	AMSDVLTV	114
6	136	LEEAHAIARHVLER	149

**Table 2:** Toxicity study of the top seven ligands produced by Dock Blaster after HTS against a drug like subset of ZINC database. Due to the toxicity effect, the removed molecules are in bold.

Molecules	Chemical Structure	Mutageic	Tumorigenic	Irritant	Reproductive Effects
*N*-(4-Methyl-2-pyridinyl)-2-(1-naphthyl)acetamide	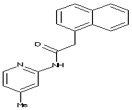	Non	Non	Non	No
3-(1,2-Diazabicyclo[2.2.2]oct-2-yl)-1-phenylpropyl acetate	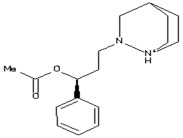	Non	Non	Medium risk of irritation	Mediumrisk of Reproductive effect
*N*-(3,5-Dimethylphenyl)-2-{4-hydroxy-2-[(1-methylethylidene)hydrazono]-2,5-dihydro-1,3-thiazol-5-yl}acetamide	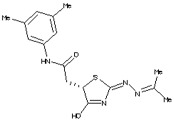	Non	Non	Non	No
(−)-Dimethyl (−)-2,3-*O*-isopropylidene-l-tartrate	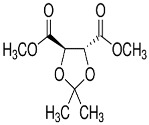	Non	Non	Non	No
1-(3-Methylphenyl)-2,5-dioxo-3-pyrrolidinyl *N*’-phenylimidothiocarbamate	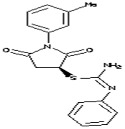	Non	Non	Non	No
Methyl (4R,5R)-5-Bromomethyl-2,2-dimethyl [1,3]dioxolane4-carboxylate	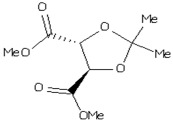	Non	Non	Non	No
tert-Butyl(2S)-2-tert-Butoxycarbonylamino-6-[3-(4-meth oxyphenyl)oxaziridin-2-yl] hexanoate	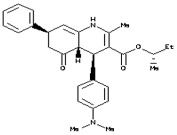	Non	Non	Non	No

**Table 3:** The drug score and very significant parameters study of the six non-toxic ligand molecules. The molecule has been chosen for docking indicated in bold.

Name of Drug	Chemical Structure	cLogP	Solubility	Mol.Wt	TPSA	Drug Likeliness	Drug Score
*N*-(4-methyl-2-pyridinyl)-2-(1-naphthyl)acetamide	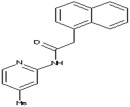	−0.54	−4.04	276	89.42	2.61	0.90
*N*-(3,5-dimethylphenyl)-2-{4-hydroxy-2-[(1-methylethylidene)hydrazono]-2,5-dihydro-1,3-thiazol-5-yl}acetamide	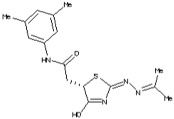	−1.27	−0.42	332	93.06	−5.79	0.67
(−)-dimethyl(−)-2,3-*O*-isopropylidene-l-tartrate	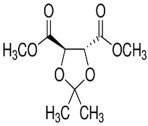	−1.68	−0.14	219	113.9	1.16	0.97
1-(3-methylphenyl)-2,5-dioxo-3-pyrrolidinyl *N*’-phenylimidothiocarbamate	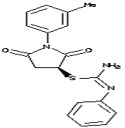	0.45	−1.28	339	93.78	1.06	0.89
Methyl (4R,5R)-5-Bromomethyl-2,2-dimethyl [1,3]dioxolane4-carboxylate	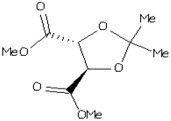	−3.02	−0.41	245	119.5	−10.75	0.39
tert-Butyl(2S)-2-tert-Butoxycarbonylamino-6-[3-(4-meth oxyphenyl)oxaziridin-2-yl] hexanoate	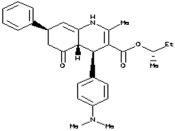	−2.17	−0.58	211	105.4	2.52	0.87

**Table 4:** The calculation of energy binding affinity and intermolecular energy of LuxS proteins with ligand by docking server.

Protein PDB: ID and Species Name	Energy Binding Affinity	Total Intermolecular Energy
LuxS (predicted model) *A. hydrophila*	−3.06 (Kcal/mol)	−4.26 kcal/mol
LuxS (5e68) *Salmonella typhi*	−2.50 (Kcal/mol)	−3.63 kcal/mol
LuxS (4XCH) *Streptococcus suis*	−1.76 (Kcal/mol)	−2.90 kcal/mol
LuxS (1VJE) *Deinococcus radiodurans*	−2.55 (Kcal/mol)	−3.69 kcal/mol

## Supplementary Materials

Supplementary materials are available online.
