# Supplementary material for: In-Silico Prediction and Modeling of the Quorum Sensing LuxS Protein and Inhibition of AI-2 Biosynthesis in Aeromonas hydrophila
Source: Molecules. 2018 Oct 12;23(10):2627. doi: 10.3390/molecules23102627 (PMC6222731; doi:10.3390/molecules23102627)

PROVE

model1.pdb

Analysis of entire structure

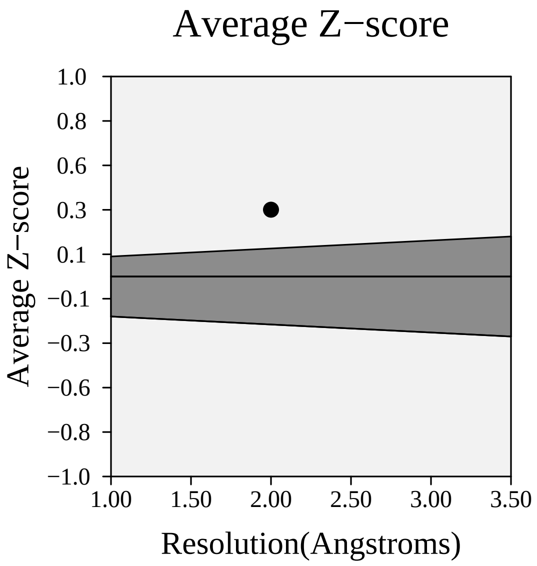

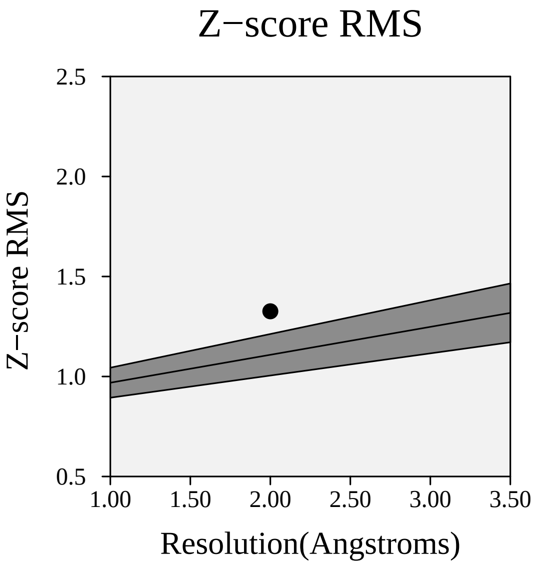

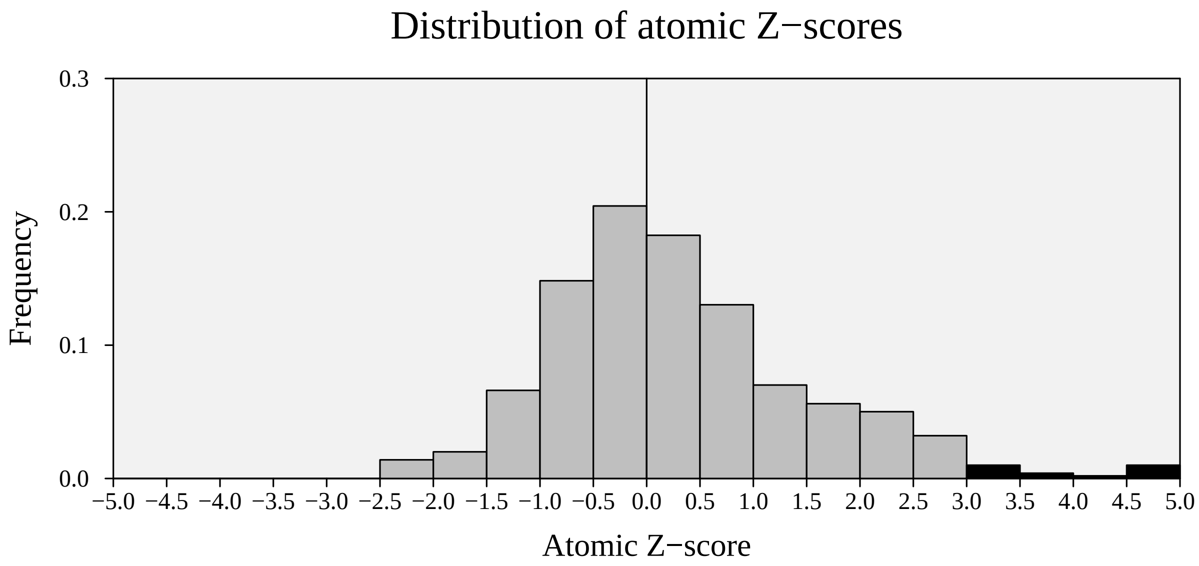

Z−score mean	0.334
Z−score stddev	1.284
Z−score RMS	1.326
# scored atoms	552
# outliers	13
% outliers	2.400

Pontius et al. (1996) J. Mol. Biol. 264, 121−136

PROVE

model1.pdb

Analysis of residues

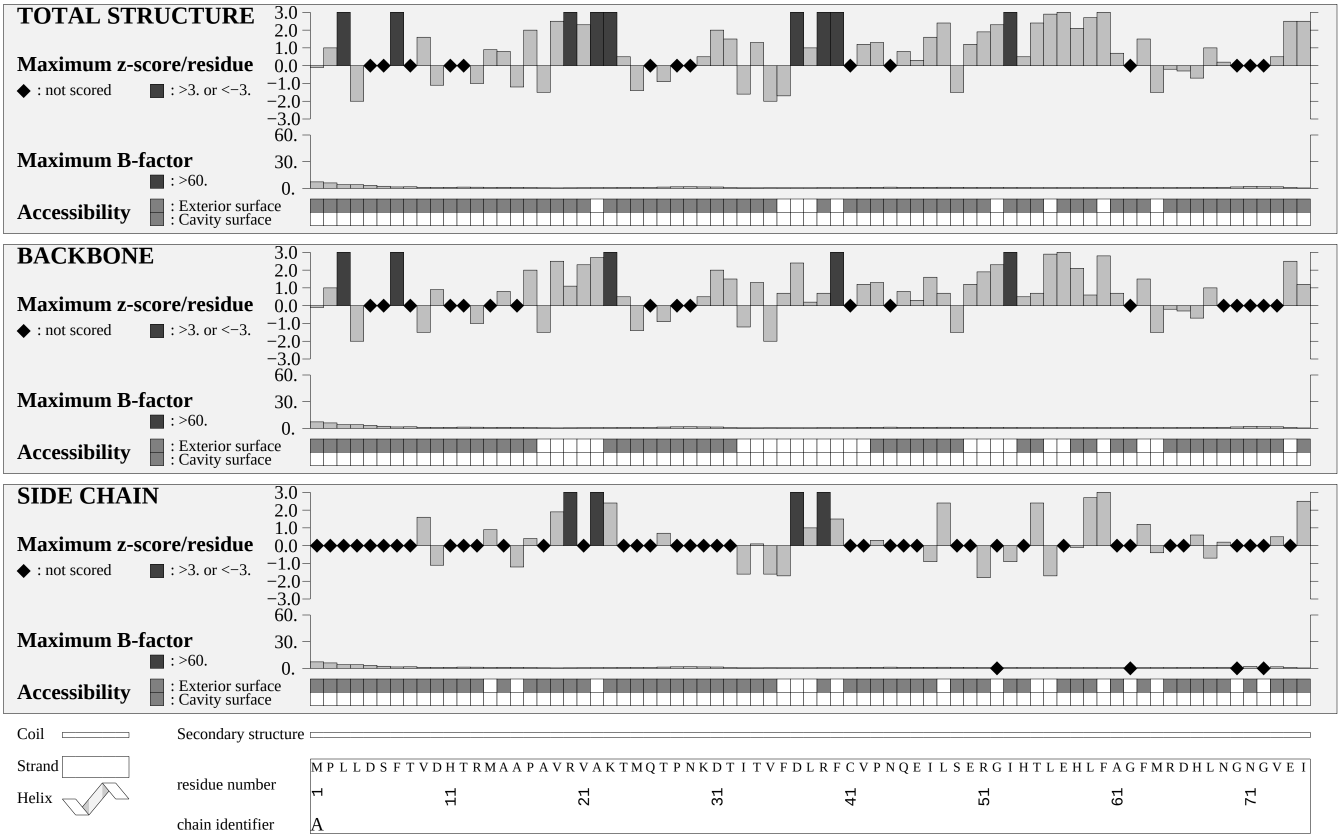

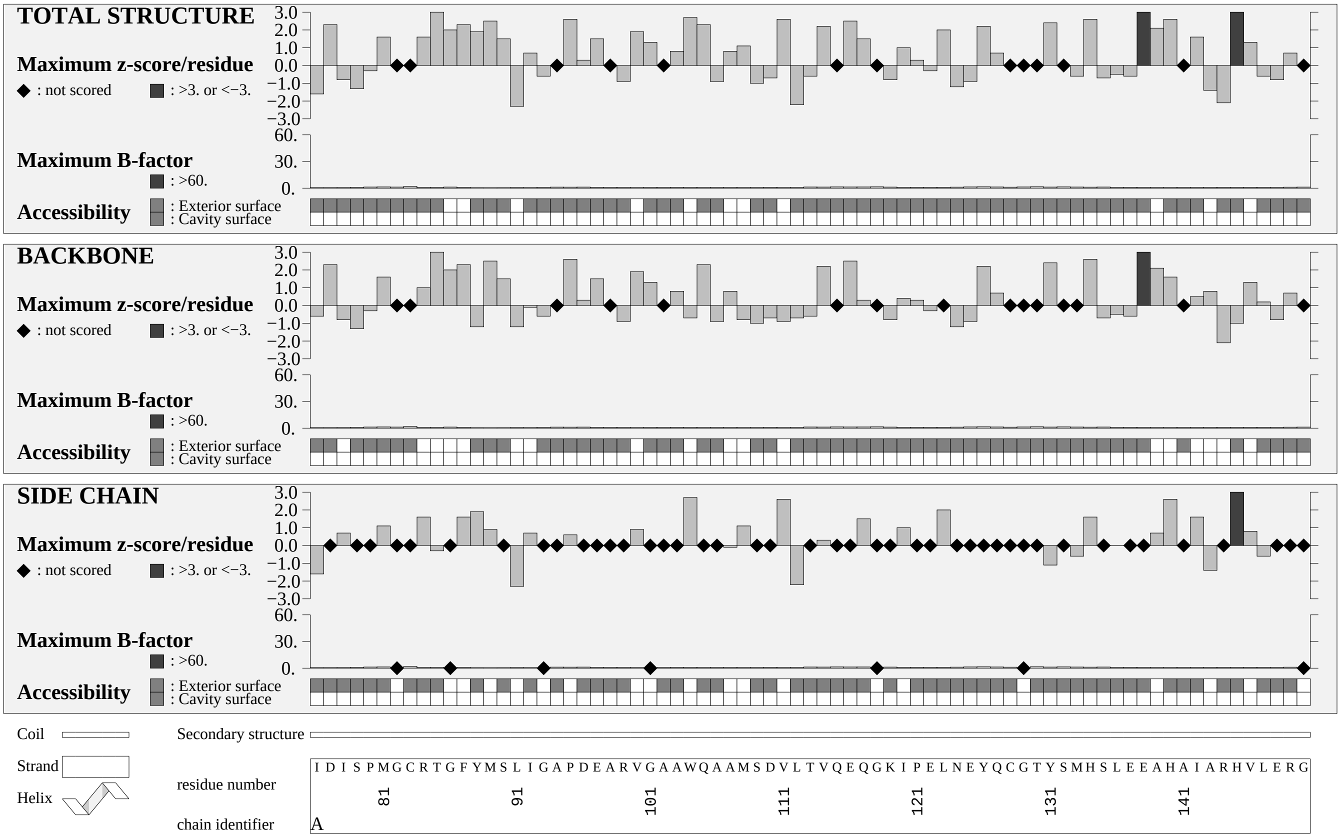

PROVE

model1.pdb

Analysis of residues (2)

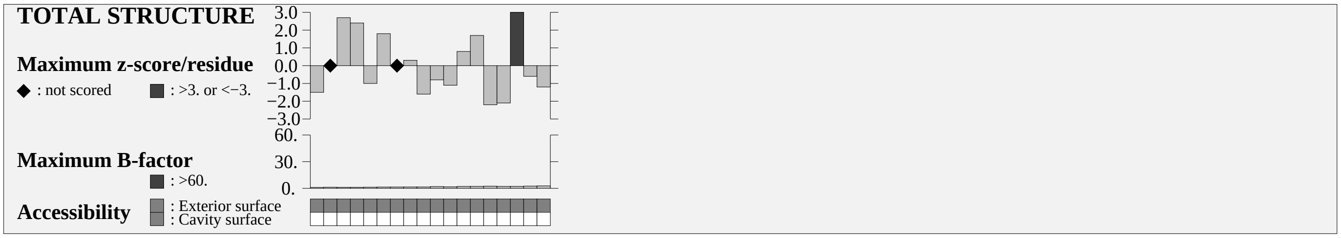

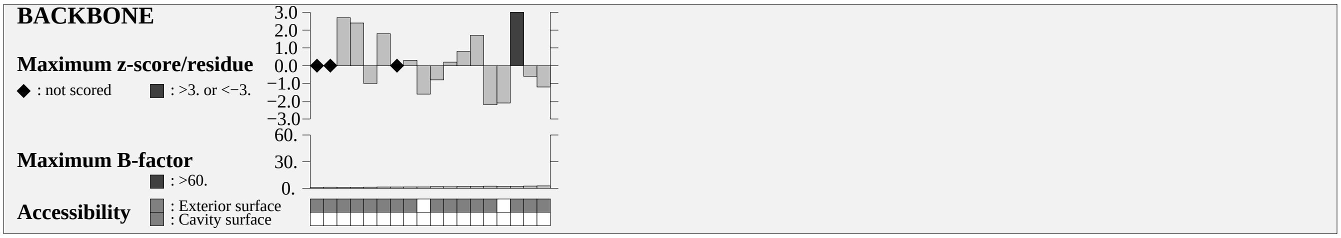

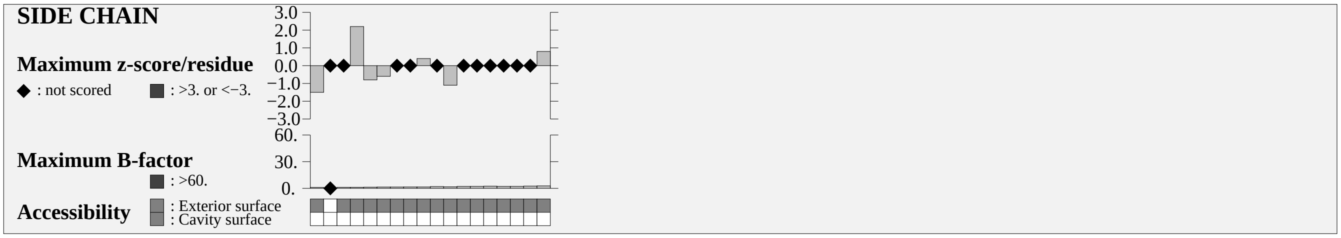

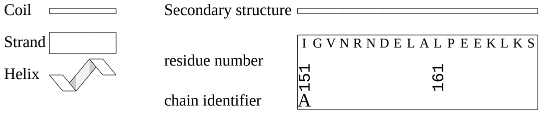

Supplement: Supplementary file 1 [file molecules-23-02627-s001.zip › Supplementary/PROVE_PLOTluxs supplementary 3.PDF]
