# Supplementary material for: In-Silico Prediction and Modeling of the Quorum Sensing LuxS Protein and Inhibition of AI-2 Biosynthesis in Aeromonas hydrophila
Source: Molecules. 2018 Oct 12;23(10):2627. doi: 10.3390/molecules23102627 (PMC6222731; doi:10.3390/molecules23102627)
Supplement: Supplementary file 1 [file molecules-23-02627-s001.zip › Supplementary/RAMPAGE luxs Supplementary 1.pdf]

RAMPAGE: Assessment of the Ramachandran Plot

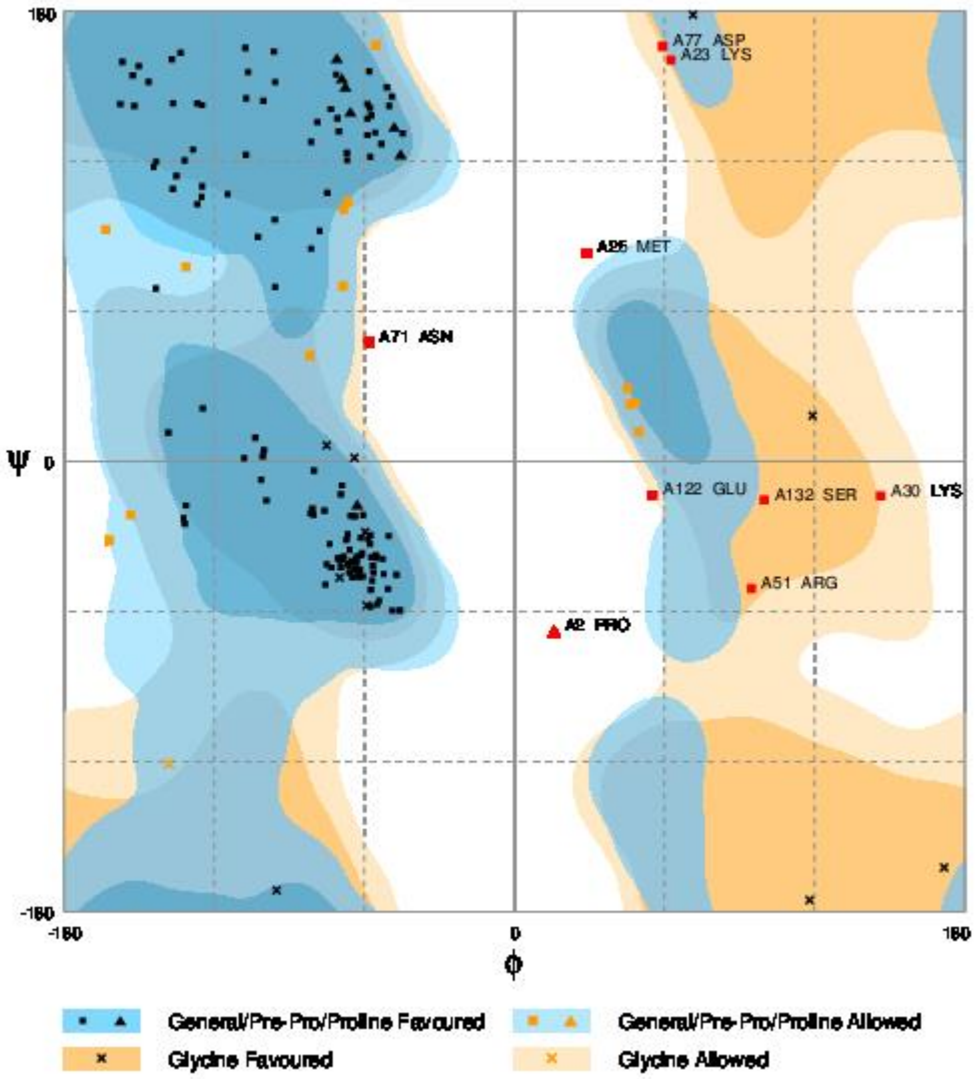

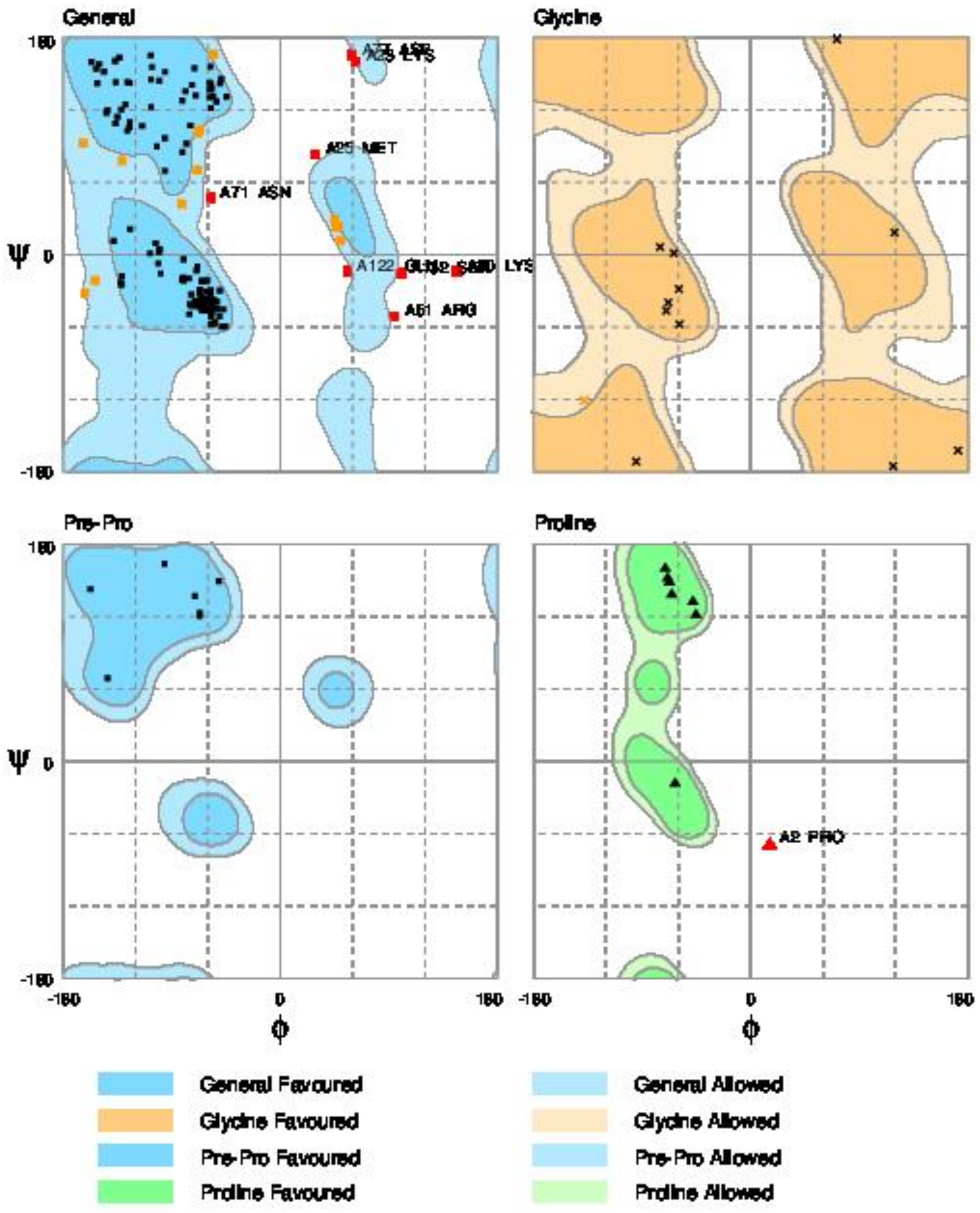
General
Glycine
Pre-Pro
Proline
A25 MET
A71 ASN
A122
LYS
A51 ARG
A2 PRO
ψ
φ
180
0
-180
General Favoured
Glycine Favoured
Pre-Pro Favoured
Proline Favoured
General Allowed
Glycine Allowed
Pre-Pro Allowed
Proline Allowed

Evaluation of residues

```
Residue [A   3 :LEU] (  49.76,   11.91) in Allowed region
Residue [A   5 :ASP] (  46.20,   22.92) in Allowed region
Residue [A  14 :MET] ( -66.75,  103.68) in Allowed region
Residue [A  24 :THR] (-161.94,  -31.53) in Allowed region
Residue [A  31 :ASP] ( -55.34,  166.25) in Allowed region
Residue [A  44 :ASN] (  48.39,   23.36) in Allowed region
Residue [A  70 :GLY] (-138.01,-120.82) in Allowed region
Residue [A  78 :ILE] (-153.41,  -21.39) in Allowed region
Residue [A  84 :ARG] (  45.19,   29.30) in Allowed region
Residue [A 114 :VAL] ( -68.20,  100.72) in Allowed region
Residue [A 123 :LEU] ( -68.53,   70.12) in Allowed region
Residue [A 133 :MET] ( -81.70,   42.29) in Allowed region
Residue [A 135 :SER] (-163.31,   92.56) in Allowed region
Residue [A 154 :ASN] (-131.18,   77.83) in Allowed region
Residue [A   2 :PRO] (  15.90,  -67.89) in Outlier region
Residue [A  23 :LYS] (  62.60,  160.43) in Outlier region
Residue [A  25 :MET] (  29.06,   83.27) in Outlier region
Residue [A  30 :LYS] ( 146.48,  -13.83) in Outlier region
Residue [A  51 :ARG] (  94.80,  -50.78) in Outlier region
Residue [A  71 :ASN] ( -58.04,   47.49) in Outlier region
Residue [A  77 :ASP] (  59.30,  165.86) in Outlier region
Residue [A 122 :GLU] (  55.55,  -13.48) in Outlier region
Residue [A 132 :SER] (  99.90,  -15.44) in Outlier region
Number of residues in favoured region    (~98.0% expected)  :  144 ( 86.2%)
Number of residues in allowed region     ( ~2.0% expected)  :   14 (  8.4%)
Number of residues in outlier region                        :    9 (  5.4%)
```
