# Supplementary material for: In-Silico Prediction and Modeling of the Quorum Sensing LuxS Protein and Inhibition of AI-2 Biosynthesis in Aeromonas hydrophila
Source: Molecules. 2018 Oct 12;23(10):2627. doi: 10.3390/molecules23102627 (PMC6222731; doi:10.3390/molecules23102627)
Supplement: Supplementary file 1 [file molecules-23-02627-s001.zip › Supplementary/Suppelenemtary 10Main-chain bond lengths 2958850_03.pdf]

PROCHECK

Main-chain bond lengths

2958850

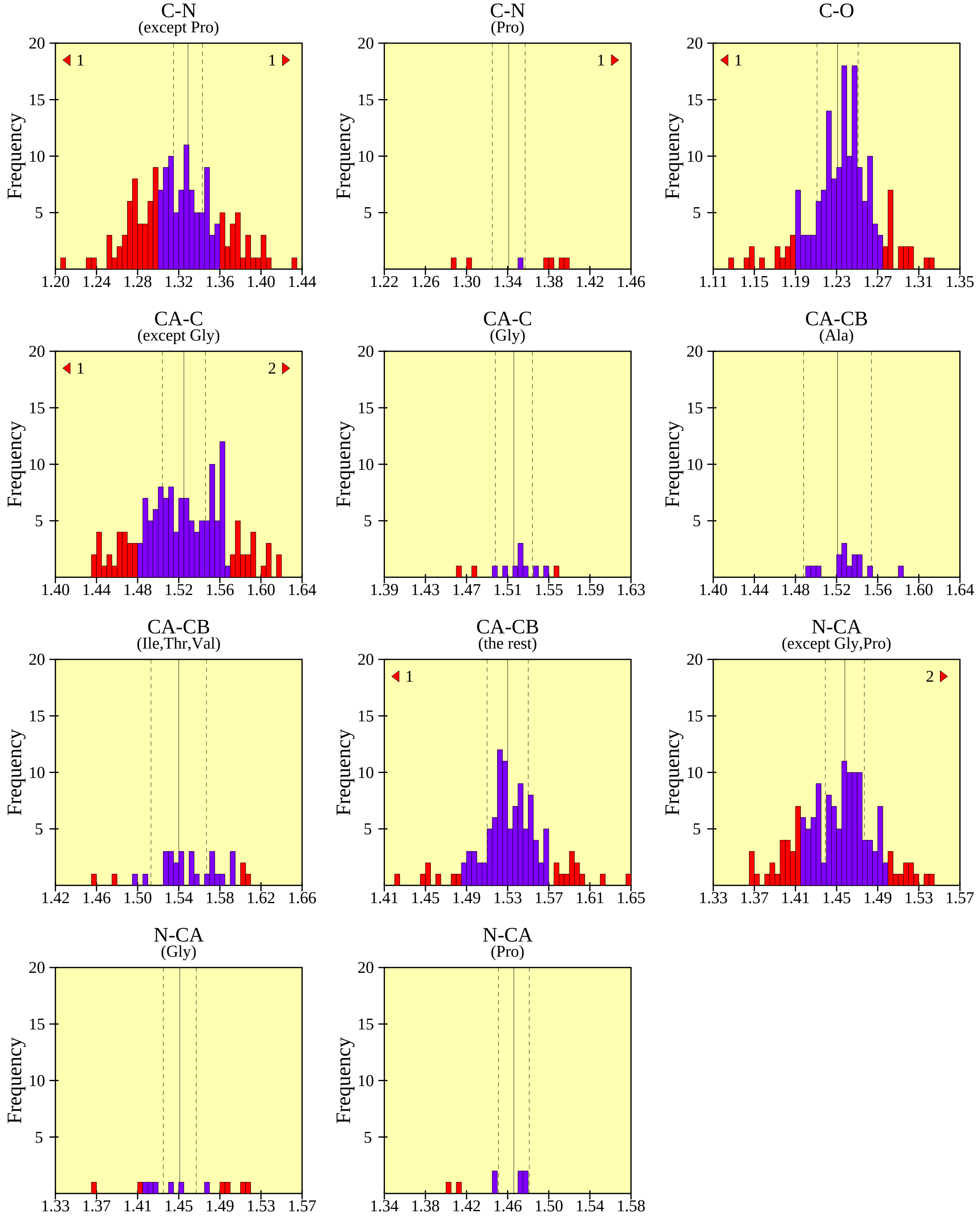

Black bars > 2.0 st. devs. from mean. ◀ or ▶ signifies data points off the graph in the direction shown.
Solid and dashed lines represent the mean and standard deviation values as per Engh & Huber small-molecule data.
