# Supplementary material for: In-Silico Prediction and Modeling of the Quorum Sensing LuxS Protein and Inhibition of AI-2 Biosynthesis in Aeromonas hydrophila
Source: Molecules. 2018 Oct 12;23(10):2627. doi: 10.3390/molecules23102627 (PMC6222731; doi:10.3390/molecules23102627)

Residue properties

2958850

a. Absolute deviation from mean Chi-1 value (excl. Pro)

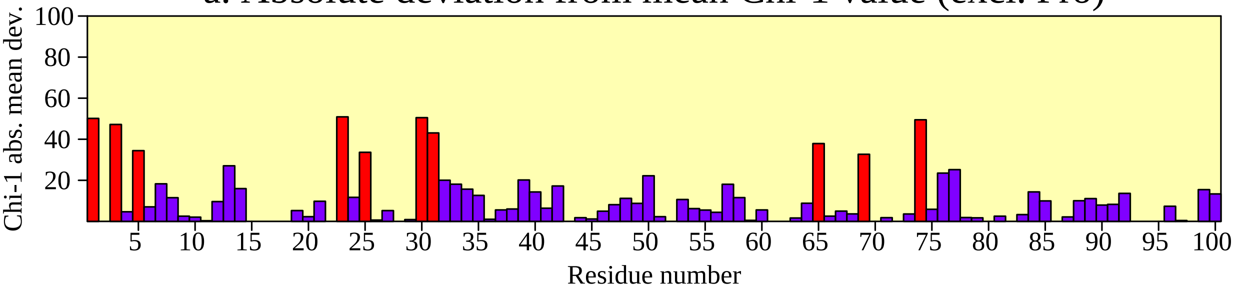

b. Absolute deviation from mean of omega torsion

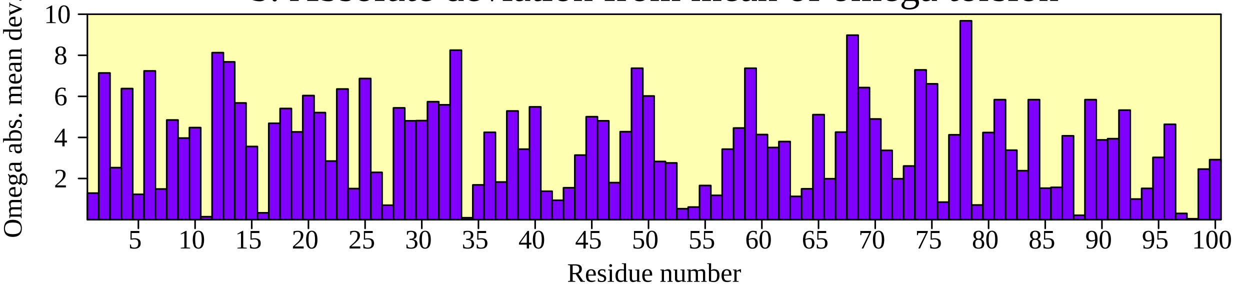

c. C-alpha chirality: abs. deviation of zeta torsion

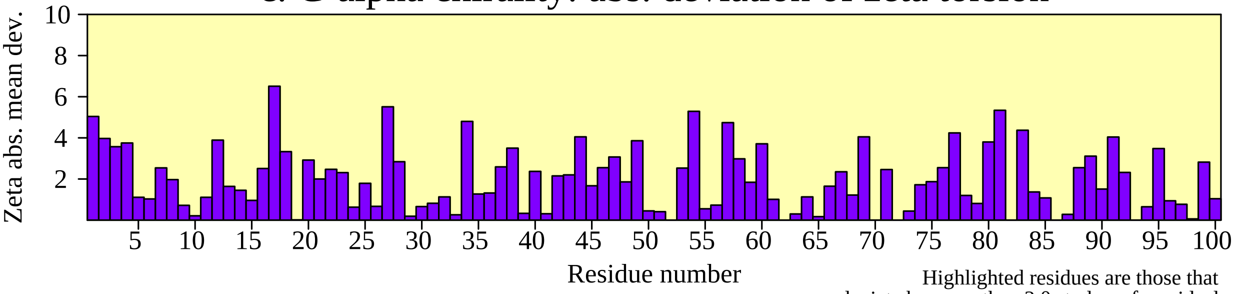

Highlighted residues are those that deviate by more than 2.0 st. devs. from ideal

d. Secondary structure & estimated accessibility

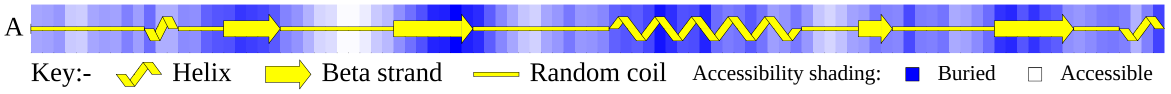

e. Sequence & Ramachandran regions

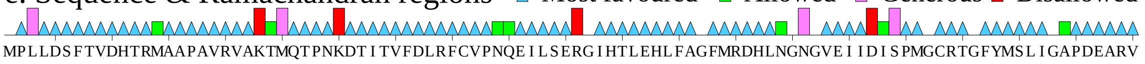

f. Max. deviation (see listing)

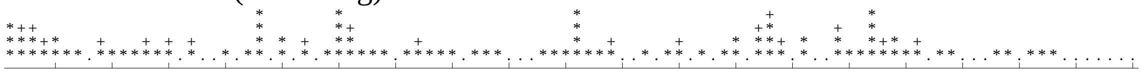

g. G-factors

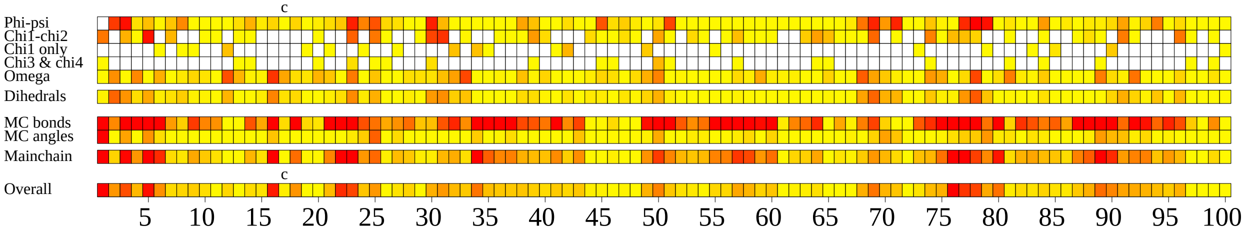

c = cis-peptide

Residue properties

2958850

a. Absolute deviation from mean Chi-1 value (excl. Pro)

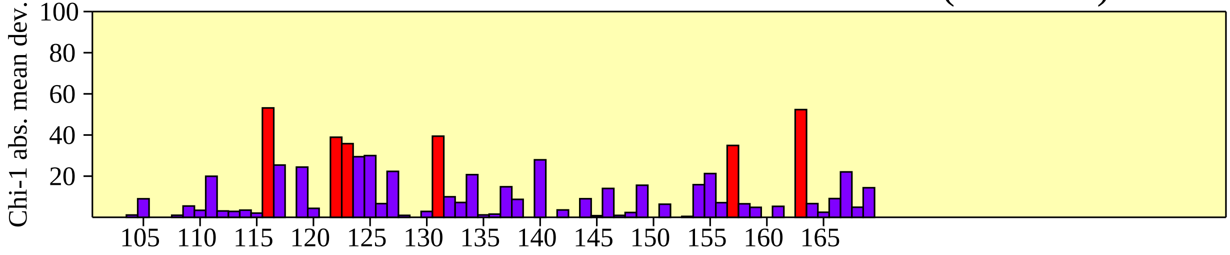

b. Absolute deviation from mean of omega torsion

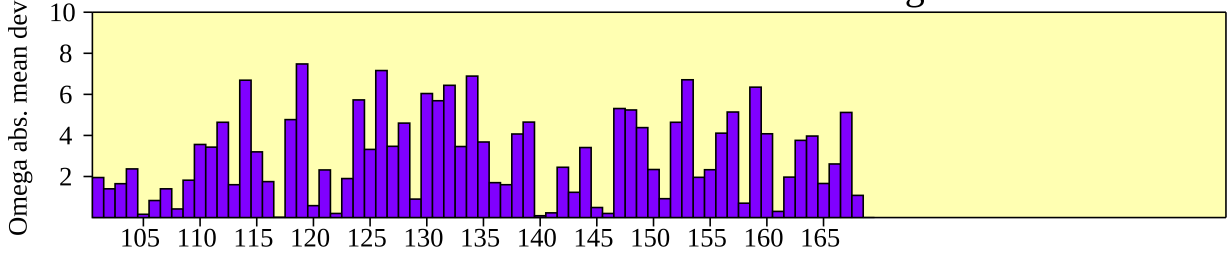

c. C-alpha chirality: abs. deviation of zeta torsion

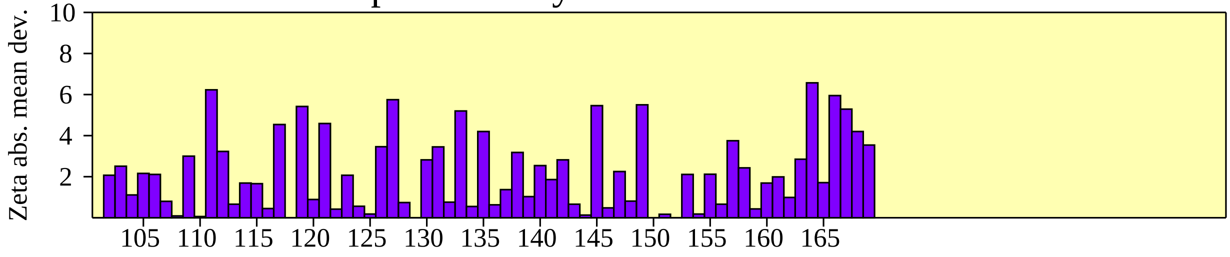

Highlighted residues are those that deviate by more than 2.0 st. devs. from ideal

d. Secondary structure & estimated accessibility

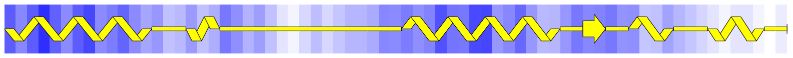

Key:- Helix Beta strand Random coil Accessibility shading: Buried Accessible

e. Sequence & Ramachandran regions

Most favoured Allowed Generous Disallowed

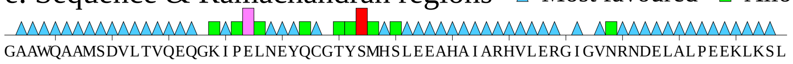

f. Max. deviation (see listing)

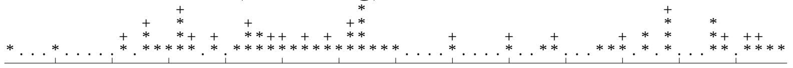

g. G-factors

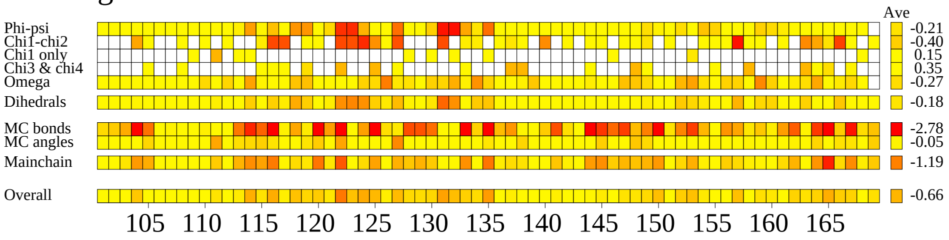

Supplement: Supplementary file 1 [file molecules-23-02627-s001.zip › Supplementary/Suppelenemtary 6Residue properties 2958850_03.pdf]
