# Supplementary material for: In-Silico Prediction and Modeling of the Quorum Sensing LuxS Protein and Inhibition of AI-2 Biosynthesis in Aeromonas hydrophila
Source: Molecules. 2018 Oct 12;23(10):2627. doi: 10.3390/molecules23102627 (PMC6222731; doi:10.3390/molecules23102627)
Supplement: Supplementary file 1 [file molecules-23-02627-s001.zip › Supplementary/Suppelenemtary 7Residue properties 2958850_03.pdf]

PROCHECK

Side-chain parameters

2958850

a. Chi-1 gauche minus

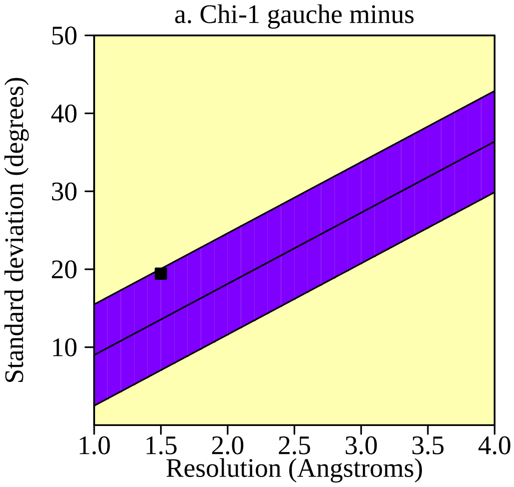

b. Chi-1 trans

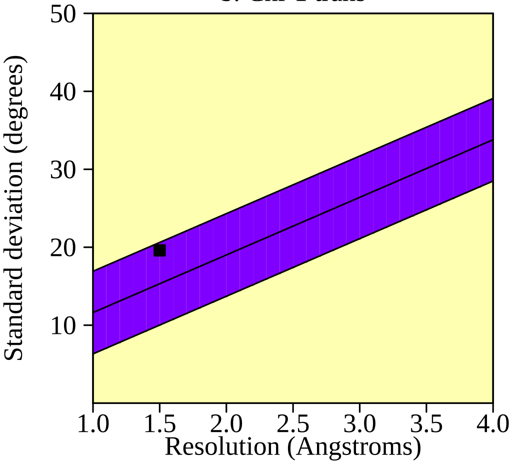

c. Chi-1 gauche plus

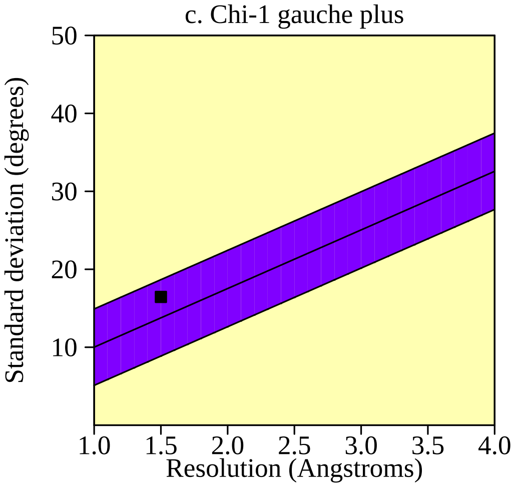

d. Chi-1 pooled standard deviation

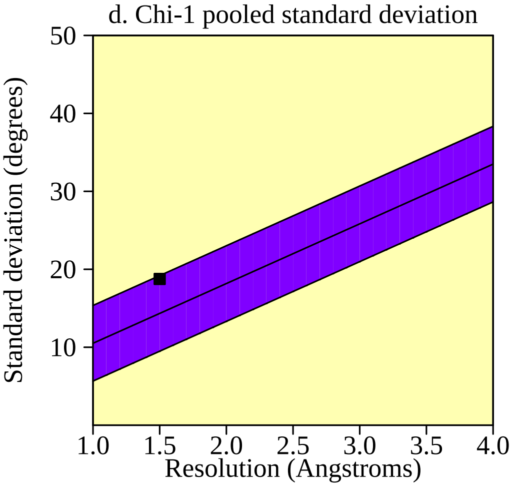

e. Standard deviation of Chi-2 trans angle

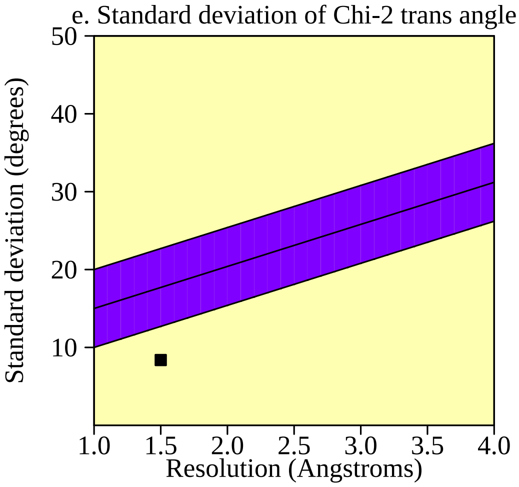

2958850

Plot statistics

Stereochemical parameter	No. of data pts	Parameter value	Comparison values Typical value	Band width	No. of band widths from mean	
a. Chi-1 gauche minus st dev	26	19.4	13.6	6.5	0.9	Inside
b. Chi-1 trans st dev	48	19.6	15.3	5.3	0.8	Inside
c. Chi-1 gauche plus st dev	60	16.5	13.8	4.9	0.5	Inside
d. Chi-1 pooled st dev	134	18.8	14.3	4.8	0.9	Inside
e. Chi-2 trans st dev	42	8.4	17.7	5.0	-1.9	BETTER

2958850_05.ps
