# Supplementary material for: In-Silico Prediction and Modeling of the Quorum Sensing LuxS Protein and Inhibition of AI-2 Biosynthesis in Aeromonas hydrophila
Source: Molecules. 2018 Oct 12;23(10):2627. doi: 10.3390/molecules23102627 (PMC6222731; doi:10.3390/molecules23102627)
Supplement: Supplementary file 1 [file molecules-23-02627-s001.zip › Supplementary/Suppelenemtary 8Main-chain parameters 2958850_03.pdf]

PROCHECK

Main-chain parameters

2958850

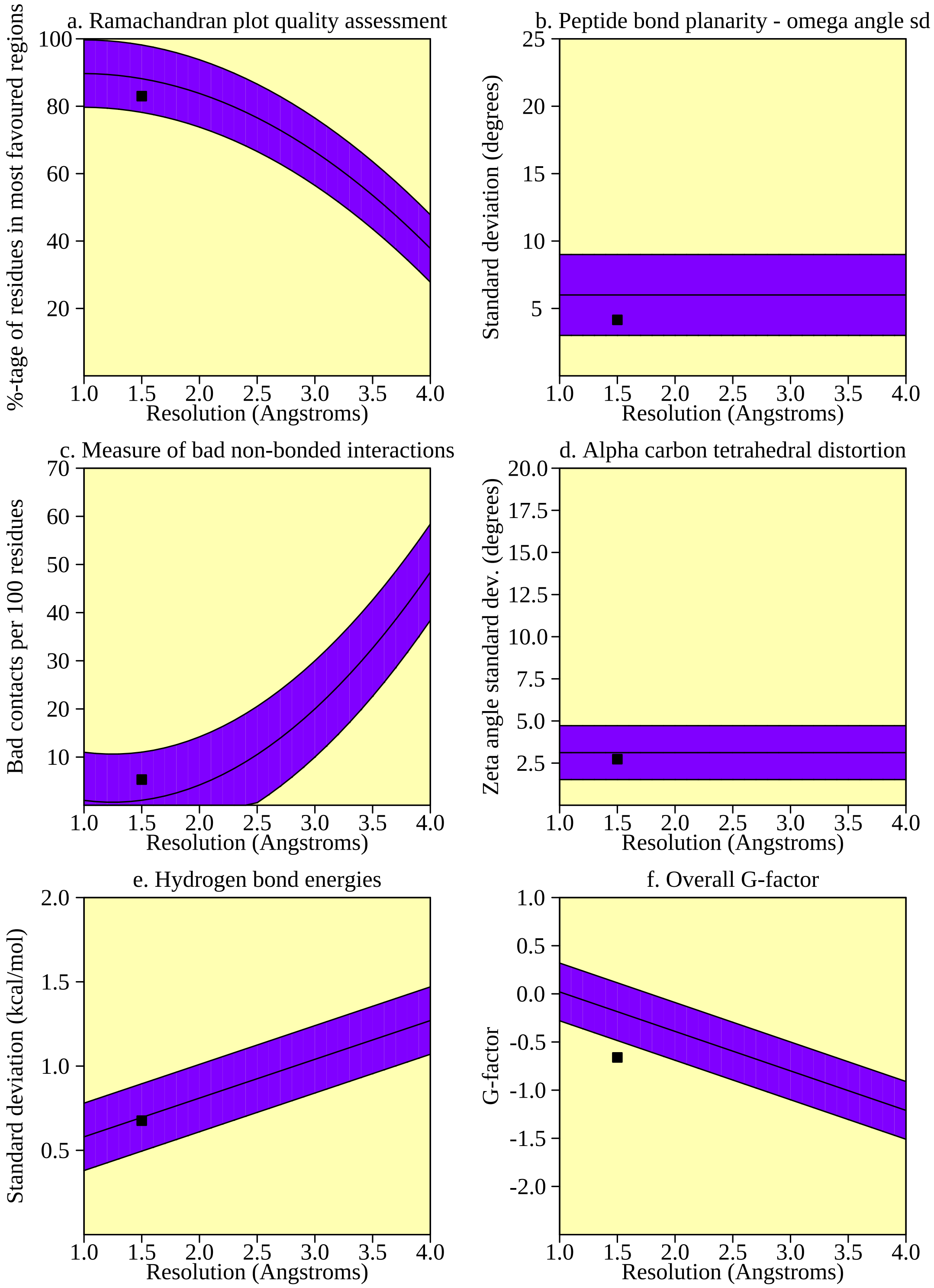

Plot statistics

Stereochemical parameter	No. of data pts	Parameter value	Comparison values Typical value	Band width	No. of band widths from mean	
a. %-tage residues in A, B, L	147	83.0	88.2	10.0	-0.5	Inside
b. Omega angle st dev	167	4.2	6.0	3.0	-0.6	Inside
c. Bad contacts / 100 residues	9	5.3	1.0	10.0	0.4	Inside
d. Zeta angle st dev	157	2.7	3.1	1.6	-0.2	Inside
e. H-bond energy st dev	105	0.7	0.7	0.2	-0.1	Inside
f. Overall G-factor	169	-0.7	-0.2	0.3	-1.6	WORSE
