# Supplementary material for: In-Silico Prediction and Modeling of the Quorum Sensing LuxS Protein and Inhibition of AI-2 Biosynthesis in Aeromonas hydrophila
Source: Molecules. 2018 Oct 12;23(10):2627. doi: 10.3390/molecules23102627 (PMC6222731; doi:10.3390/molecules23102627)
Supplement: Supplementary file 1 [file molecules-23-02627-s001.zip › Supplementary/Suppelenemtary 9Distorted geometry 2958850_03.pdf]

PROCHECK

Distorted geometry

2958850

Main-chain bond lengths

C	1.231	O	0.185	1.046	A Met 1
CA	1.530	CB	0.077	1.453	A Met 1
N	1.458	CA	0.059	1.399	A Met 1
C	1.231	O	0.073	1.158	A Pro 2
N	1.458	CA	0.119	1.577	A Leu 3
C	1.329	N	0.057	1.272	A Leu 3 - A Leu 4
C	1.231	O	0.102	1.129	A Leu 4
CA	1.530	CB	0.120	1.650	A Asp 5
N	1.458	CA	0.149	1.607	A Asp 5
CA	1.525	C	0.080	1.445	A Ser 6
CA	1.530	CB	0.082	1.448	A Ser 6
C	1.329	N	0.063	1.266	A Ser 6 - A Phe 7
C	1.231	O	0.066	1.297	A Val 9
N	1.458	CA	0.059	1.399	A Val 9
N	1.458	CA	0.053	1.405	A Asp 10
C	1.231	O	0.061	1.170	A His 11
CA	1.525	C	0.055	1.580	A Met 14
CA	1.530	CB	0.066	1.464	A Met 14
CA	1.525	C	0.066	1.591	A Ala 15
CA	1.525	C	0.069	1.594	A Ala 16
C	1.341	N	0.126	1.467	A Ala 16 - A Pro 17
C	1.231	O	0.065	1.296	A Ala 18
CA	1.525	C	0.052	1.577	A Ala 18
C	1.329	N	0.074	1.403	A Ala 18 - A Val 19
CA	1.525	C	0.079	1.446	A Val 21
C	1.329	N	0.065	1.264	A Val 21 - A Ala 22
CA	1.521	CB	0.063	1.584	A Ala 22
N	1.458	CA	0.069	1.389	A Ala 22
C	1.329	N	0.119	1.210	A Ala 22 - A Lys 23
CA	1.525	C	0.181	1.706	A Lys 23
CA	1.530	CB	0.051	1.581	A Lys 23
C	1.329	N	0.061	1.268	A Thr 24 - A Met 25
CA	1.525	C	0.080	1.605	A Met 25
C	1.341	N	0.057	1.398	A Thr 27 - A Pro 28
C	1.329	N	0.057	1.386	A Pro 28 - A Asn 29
CA	1.525	C	0.064	1.461	A Asn 29
N	1.458	CA	0.057	1.515	A Asp 31
C	1.329	N	0.053	1.382	A Asp 31 - A Thr 32
C	1.231	O	0.069	1.300	A Thr 32
C	1.329	N	0.051	1.380	A Thr 32 - A Ile 33
CA	1.540	CB	0.054	1.594	A Thr 34
C	1.329	N	0.117	1.446	A Thr 34 - A Val 35
C	1.329	N	0.092	1.237	A Val 35 - A Phe 36
C	1.231	O	0.094	1.324	A Phe 36
N	1.458	CA	0.059	1.399	A Phe 36
N	1.458	CA	0.085	1.373	A Asp 37
C	1.231	O	0.051	1.282	A Leu 38
CA	1.530	CB	0.055	1.585	A Leu 38
N	1.458	CA	0.058	1.400	A Leu 38
CA	1.525	C	0.056	1.469	A Arg 39
CA	1.525	C	0.050	1.575	A Phe 40
N	1.458	CA	0.071	1.529	A Phe 40
C	1.231	O	0.051	1.282	A Cys 41
C	1.329	N	0.065	1.394	A Cys 41 - A Val 42
C	1.231	O	0.053	1.284	A Val 42
CA	1.525	C	0.073	1.452	A Pro 43
N	1.466	CA	0.054	1.412	A Pro 43
N	1.458	CA	0.053	1.405	A Ser 49
C	1.329	N	0.064	1.265	A Ser 49 - A Glu 50
C	1.231	O	0.073	1.304	A Glu 50
CA	1.530	CB	0.062	1.592	A Glu 50
C	1.231	O	0.087	1.318	A Arg 51
C	1.329	N	0.061	1.390	A Arg 51 - A Gly 52
CA	1.516	C	0.052	1.464	A Gly 52
CA	1.540	CB	0.065	1.605	A Ile 53
CA	1.530	CB	0.072	1.602	A His 54

Distorted geometry
2958850

Main-chain bond lengths (contd)

Atom 1	Ideal	Atom 2	Sigma	Observed	Residue(s)
CA	1.540	CB	0.063	1.477	A Thr 55
C	1.329	N	0.077	1.252	A Thr 55 - A Leu 56
C	1.231	O	0.083	1.148	A Leu 56
CA	1.525	C	0.050	1.475	A Leu 56
N	1.458	CA	0.054	1.404	A Leu 56
CA	1.525	C	0.086	1.439	A Glu 57
CA	1.530	CB	0.066	1.596	A Glu 57
C	1.329	N	0.071	1.400	A Glu 57 - A His 58
CA	1.525	C	0.060	1.465	A His 58
CA	1.530	CB	0.066	1.596	A His 58
C	1.329	N	0.071	1.258	A His 58 - A Leu 59
CA	1.525	C	0.053	1.577	A Leu 59
C	1.329	N	0.057	1.272	A Leu 59 - A Phe 60
CA	1.525	C	0.059	1.465	A Phe 60
N	1.458	CA	0.070	1.388	A Phe 60
C	1.329	N	0.057	1.272	A Phe 63 - A Met 64
N	1.458	CA	0.090	1.368	A Met 64
CA	1.525	C	0.079	1.604	A Leu 68
CA	1.525	C	0.064	1.589	A Asn 69
N	1.458	CA	0.052	1.510	A Asn 69
CA	1.525	C	0.088	1.437	A Glu 74
CA	1.525	C	0.064	1.461	A Ile 75
CA	1.540	CB	0.051	1.591	A Ile 75
C	1.329	N	0.075	1.254	A Ile 75 - A Ile 76
CA	1.525	C	0.140	1.665	A Ile 76
CA	1.540	CB	0.068	1.608	A Ile 76
N	1.458	CA	0.051	1.407	A Ile 76
C	1.329	N	0.135	1.194	A Ile 76 - A Asp 77
CA	1.525	C	0.091	1.616	A Asp 77
C	1.329	N	0.095	1.234	A Asp 77 - A Ile 78
C	1.231	O	0.053	1.283	A Ile 78
CA	1.525	C	0.069	1.594	A Ile 78
C	1.329	N	0.076	1.405	A Ile 78 - A Ser 79
N	1.458	CA	0.055	1.513	A Ser 79
CA	1.525	C	0.146	1.379	A Pro 80
C	1.329	N	0.052	1.277	A Pro 80 - A Met 81
N	1.451	CA	0.064	1.515	A Gly 82
C	1.329	N	0.056	1.385	A Cys 83 - A Arg 84
CA	1.525	C	0.080	1.605	A Arg 84
C	1.231	O	0.060	1.291	A Thr 85
C	1.329	N	0.050	1.379	A Thr 85 - A Gly 86
C	1.231	O	0.059	1.290	A Phe 87
N	1.458	CA	0.079	1.536	A Phe 87
CA	1.525	C	0.081	1.444	A Tyr 88
CA	1.530	CB	0.063	1.593	A Tyr 88
C	1.329	N	0.059	1.270	A Tyr 88 - A Met 89
C	1.231	O	0.088	1.143	A Met 89
CA	1.525	C	0.081	1.444	A Met 89
C	1.329	N	0.102	1.431	A Met 89 - A Ser 90
N	1.458	CA	0.088	1.370	A Ser 90
C	1.329	N	0.075	1.254	A Ser 90 - A Leu 91
CA	1.525	C	0.067	1.458	A Leu 91
N	1.458	CA	0.062	1.395	A Ile 92
C	1.329	N	0.067	1.396	A Ile 92 - A Gly 93
C	1.231	O	0.082	1.149	A Gly 93
N	1.458	CA	0.055	1.403	A Ala 94
C	1.341	N	0.051	1.290	A Ala 94 - A Pro 95
CA	1.525	C	0.053	1.472	A Pro 95
N	1.466	CA	0.063	1.403	A Pro 95
CA	1.525	C	0.057	1.582	A Asp 96
CA	1.530	CB	0.050	1.480	A Asp 96
C	1.329	N	0.050	1.279	A Arg 99 - A Val 100
C	1.329	N	0.050	1.279	A Ala 103 - A Trp 104
C	1.329	N	0.077	1.406	A Trp 104 - A Gln 105
CA	1.525	C	0.057	1.468	A Gln 105
CA	1.540	CB	0.065	1.605	A Val 114

Distorted geometry
2958850

Main-chain bond lengths (contd)

Bond	Ideal	Difference	Actual	Residue
N - CA	1.458	0.059	1.517	A Val 114
CA - C	1.525	0.061	1.586	A Gln 115
C - O	1.231	0.051	1.282	A Glu 116
N - CA	1.458	0.086	1.544	A Glu 116
CA - C	1.525	0.075	1.450	A Ile 120
CA - CB	1.540	0.051	1.591	A Ile 120
N - CA	1.458	0.067	1.391	A Ile 120
C - O	1.231	0.054	1.176	A Pro 121
CA - C	1.525	0.066	1.591	A Glu 122
N - CA	1.458	0.066	1.523	A Glu 122
N - CA	1.458	0.065	1.523	A Glu 125
C - N	1.329	0.058	1.271	A Glu 125 - A Tyr 126
CA - C	1.525	0.091	1.616	A Cys 128
N - CA	1.451	0.064	1.515	A Gly 129
CA - C	1.525	0.083	1.608	A Thr 130
N - CA	1.458	0.091	1.367	A Met 133
CA - C	1.525	0.055	1.470	A Ser 135
CA - CB	1.530	0.108	1.422	A Ser 135
CA - C	1.525	0.061	1.464	A Ala 141
CA - C	1.525	0.082	1.442	A Arg 144
CA - CB	1.530	0.094	1.624	A His 145
CA - C	1.525	0.058	1.467	A Val 146
CA - CB	1.540	0.080	1.460	A Val 146
N - CA	1.458	0.055	1.403	A Leu 147
C - N	1.329	0.055	1.274	A Leu 147 - A Glu 148
N - CA	1.451	0.083	1.368	A Gly 150
C - N	1.329	0.052	1.277	A Val 153 - A Asn 154
C - O	1.231	0.059	1.172	A Asn 156
C - N	1.341	0.051	1.392	A Leu 161 - A Pro 162
C - N	1.329	0.055	1.274	A Pro 162 - A Glu 163
CA - CB	1.530	0.061	1.590	A Glu 164
C - N	1.329	0.051	1.278	A Glu 164 - A Lys 165
CA - CB	1.530	0.134	1.396	A Lys 165
N - CA	1.458	0.076	1.382	A Lys 165
CA - CB	1.530	0.079	1.451	A Lys 167
CA - C	1.525	0.056	1.581	A Ser 168
CA - C	1.525	0.051	1.576	A Leu 169

Bonds differing by > 0.05A from small-molecule values. Values shown: "ideal", difference, actual

Main-chain bond angles

Angle	Ideal	Actual	Diff	Residues
CA - C - N	116.2	126.7	10.5	A Met 1 - A Pro 2

Bond angles differing by > 10.0 degrees from small-molec values. Values shown: "ideal", actual, diff.

Distorted geometry
2958850

Planar groups

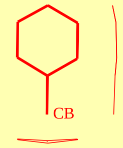

0.058
A Phe 7

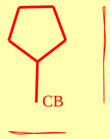

0.032
A His 54

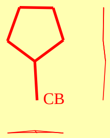

0.034
A His 58

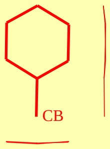

0.031
A Phe 60

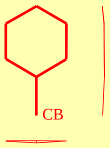

0.032
A Phe 87

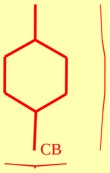

0.074
A Tyr 88

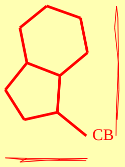

0.049
A Trp 104

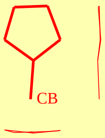

0.042
A His 140

Sidechains with RMS dist. from planarity > 0.03A for rings, or > 0.02A otherwise. Value shown is RMS dist.
