# Supplementary material for: In-Silico Prediction and Modeling of the Quorum Sensing LuxS Protein and Inhibition of AI-2 Biosynthesis in Aeromonas hydrophila
Source: Molecules. 2018 Oct 12;23(10):2627. doi: 10.3390/molecules23102627 (PMC6222731; doi:10.3390/molecules23102627)
Supplement: Supplementary file 1 [file molecules-23-02627-s001.zip › Supplementary/supplementary 4RMS distances from planarity2958850_09.pdf]

PROCHECK

RMS distances from planarity

2958850

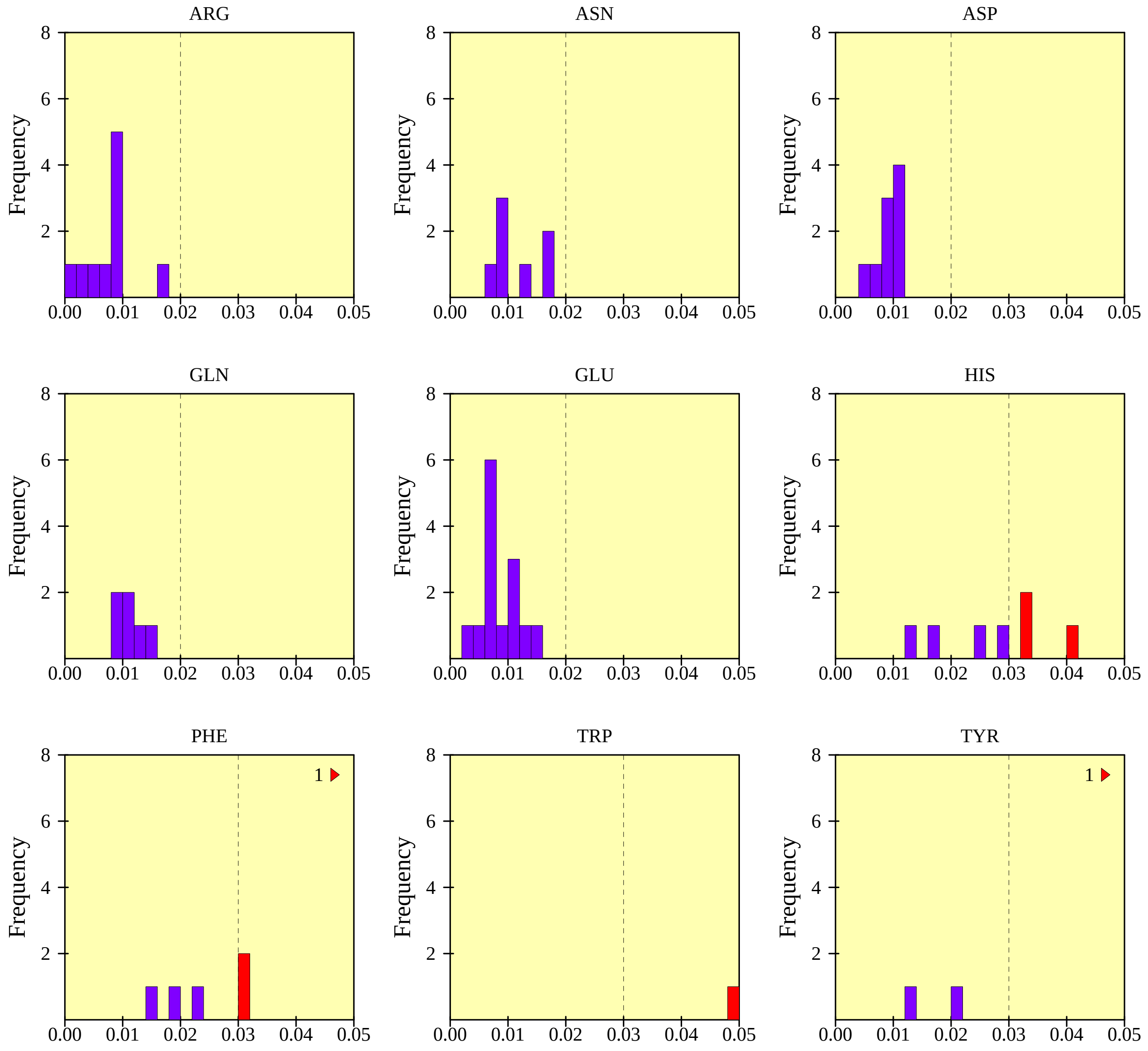

Histograms showing RMS distances of planar atoms from best-fit plane.
Black bars indicate large deviations from planarity: RMS dist > 0.03 for rings, and > 0.02 otherwise.

signifies data points off the graph in the direction shown.
