# Supplementary material for: In-Silico Prediction and Modeling of the Quorum Sensing LuxS Protein and Inhibition of AI-2 Biosynthesis in Aeromonas hydrophila
Source: Molecules. 2018 Oct 12;23(10):2627. doi: 10.3390/molecules23102627 (PMC6222731; doi:10.3390/molecules23102627)
Supplement: Supplementary file 1 [file molecules-23-02627-s001.zip › Supplementary/Supplementary figures and tables LuxS.docx]

**Supplementary Table.1** Molecular profile of LuxS protein

| **No.** | **Properties** | **LuxS** |
| --- | --- | --- |
| 1 | No.of amino acids | 169 |
| 2 | Molecular weight | 18.79kDa |
| 3 | Formula | C_821_H_1304_N_232_O_250_S_11_ |
| 4 | Total no.of atoms | 2618 |
| 5 | I so electric point PI | 5.13 |
| 6 | Ext.coeffiecient | 9970 |
| 7 | Half-life estimation | 30hrs (mammalian reticulocyte, *in vitro*)  >20hrs (yeast*, in vivo*)  >10hrs (*E.coli, in vivo*) |
| 8 | Aliphatic index | 87.75 |
| 9 | Grand average hydrophobicity (GRAVY) | -0.179 moderately hydrophilic |
| 10 | CELLO prediction localization scores:  Cytoplasmic | 4.504* |
| 11 | Instability index (protein is stable) | 39.87 |

**Supplementary Table 2. Obtained results from the docking of (−)-Dimethyl 2,3-O-isopropylidene-L-tartrate into predicted LuxS model interaction by Swiss Dock**

**Receptor No. of Swiss dock cluster cluster rank Full fitness (kcal/mol) Estimated ΔG (kcal/mol)**

LuxS protein 250 runs 0 -674.81 -6.39

1 -674.79 -6.40

2 -674.65 -6.30

3 -674.38 -6.31

4 -674.06 -6.27

5 -664.15 -6.44

6 -661.75 -6.29

**S Figure 1**


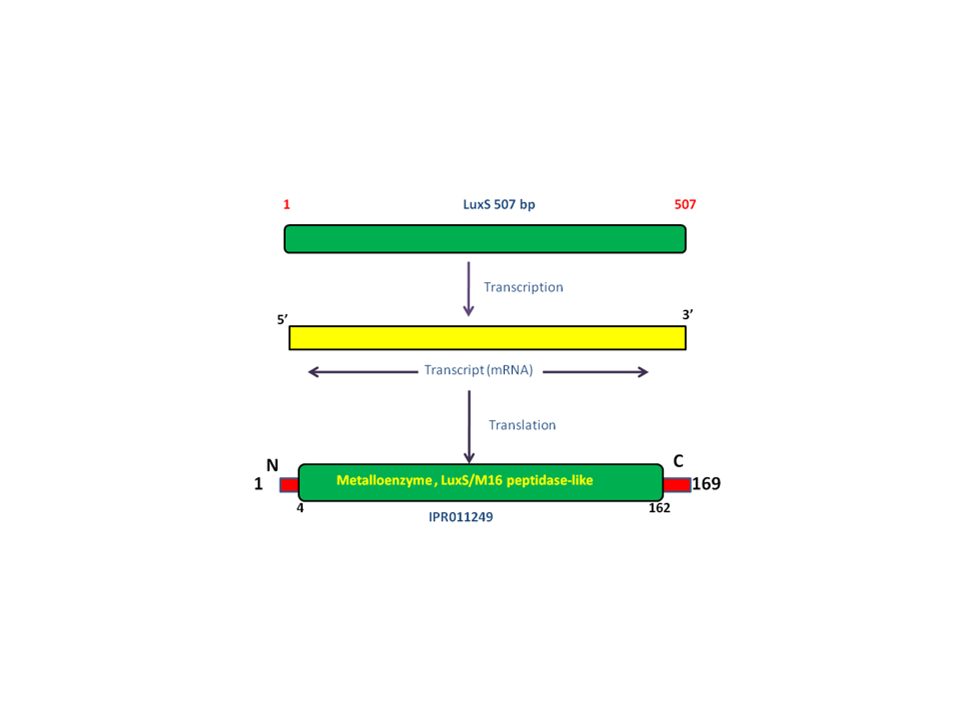


**Supplementary Figure 1.** The architecture of *LuxS* gene. The *LuxS* gene (green 507bp) followed by transcription and translation is expressed into metalloenzyme, LuxS/M16 peptidase like functional domain (4-162) was found from N-C terminal represented at the bottom in (green) with InterPro protein families database ID.IPR011249.

**S Figure 2**


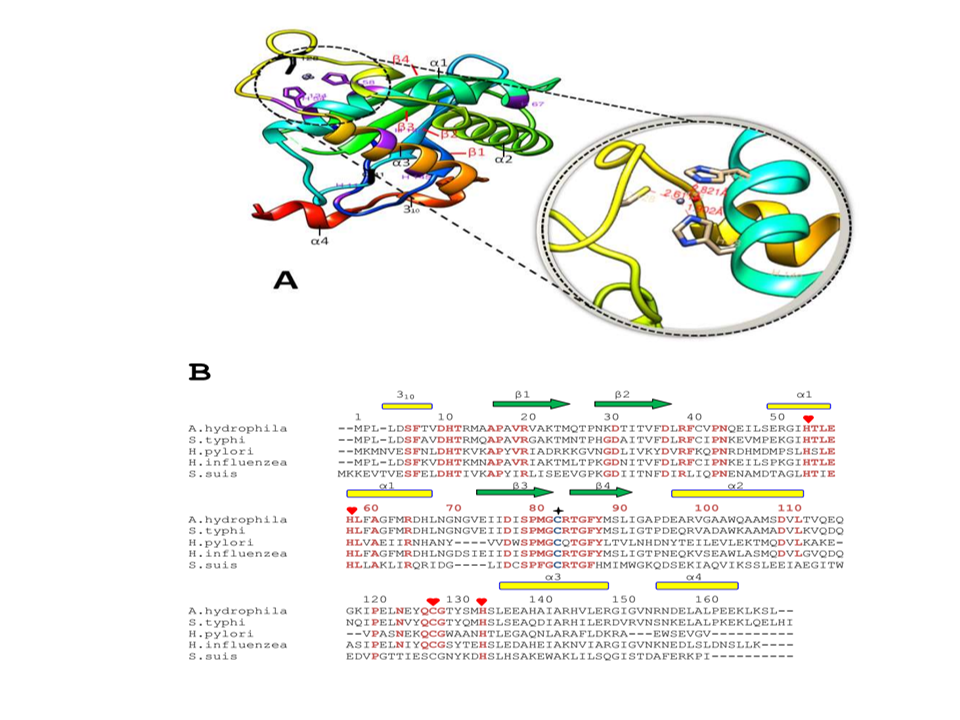


**Supplementary Figure 2.** Predicted Zn-ligand cluster and the interatomic distances of the active site, and sequence alignment of LuxS. **(A):** Monomer topological structure of LuxS displays the structures of helices, sheets and the Zinc-binding sites (*top left*). Helices and sheets are shown with α1-4 and β1-4 respectively. Zinc is illustrated into a black sphere and the protein ligands, His-54, 58 and Cys128 are labeled with sticks and ring shapes. **(B)** LuxS Sequence alignment of LuxS from selected organisms and conserved residues among sequences are shown in red. Zn ligand binding residues are labeled with red heart but cysteine is oxidized indicated in the black star.

**
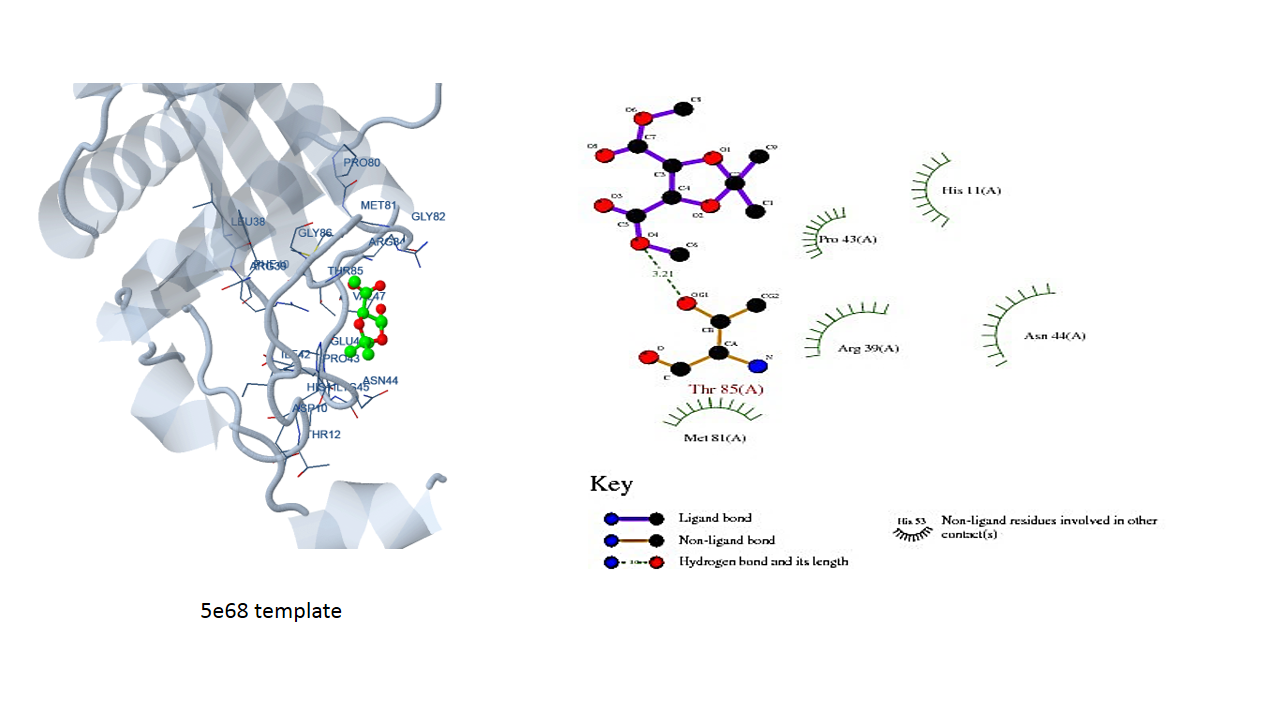
**

**(B)**

**Supplementary Figure 3. (A)** 5e68 homologous to LuxS, ligand –protein complex docked by dock server **(B)** Schematic illustration interaction of dimethyl (-)-2,3-O-isopropylidene-L-tartrate ligand molecule with 5e68 protein.

**
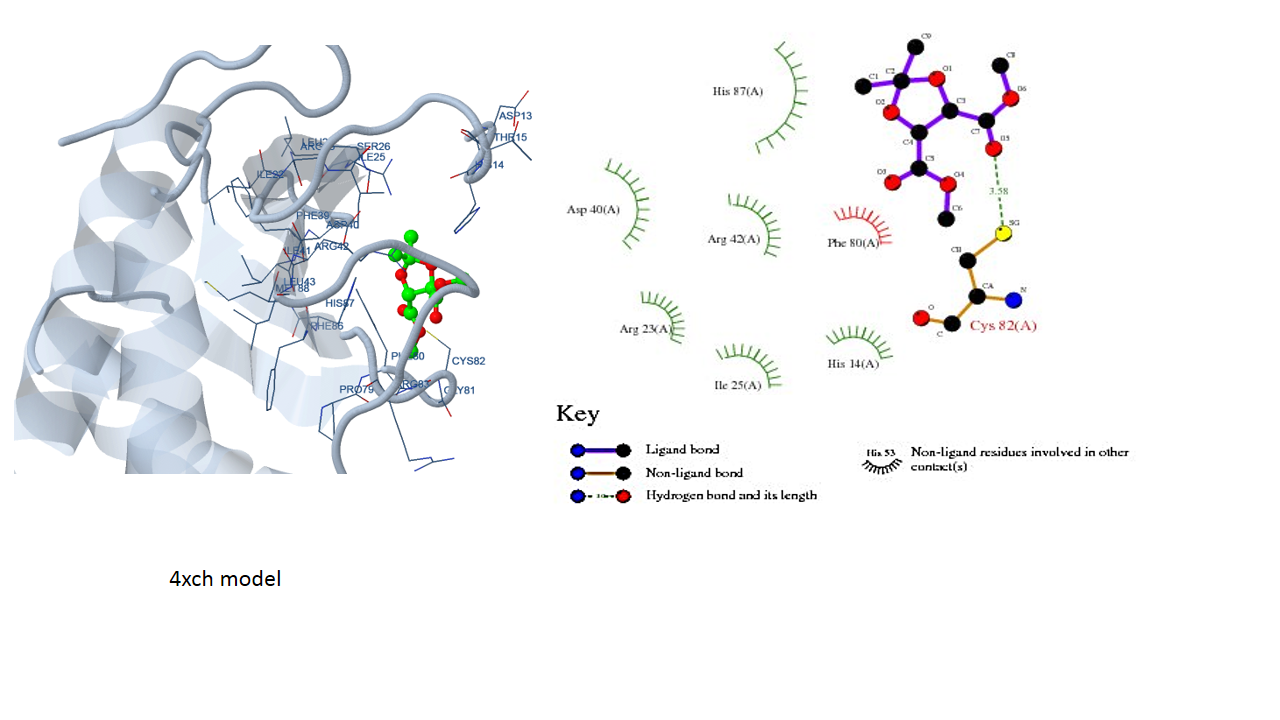
**

**(B)**

**Supplementary Figure 4. (A)** 4XCH homologous to LuxS, ligand –protein complex docked by dock server **(B)** Schematic illustration interaction of dimethyl (-)-2,3-O-isopropylidene-L-tartrate ligand molecule with 4XCH protein.

**
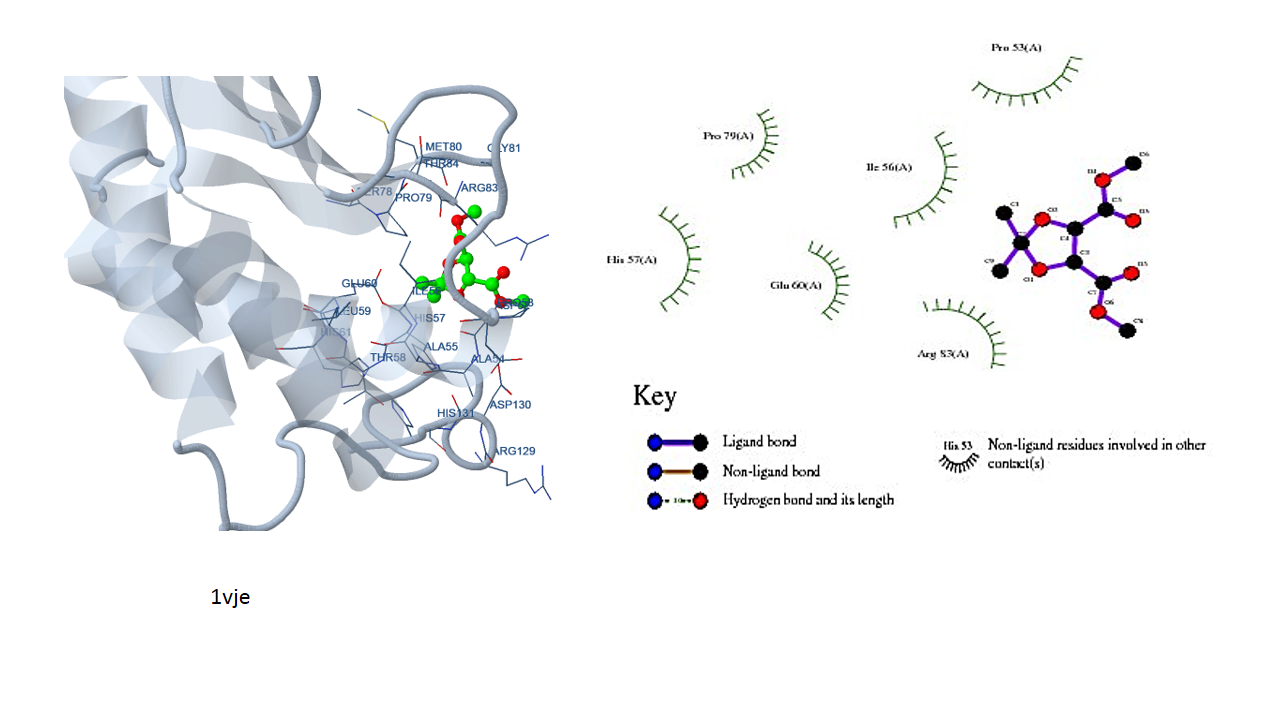
**

**(B)**

**Supplementary Figure 5. (A)** 1VJE homologous to LuxS, ligand –protein complex docked by dock server **(B)** Schematic illustration interaction of dimethyl (-)-2,3-O-isopropylidene-L-tartrate ligand molecule with 1VJE protein.
