# Supplementary material for: In-Silico Prediction and Modeling of the Quorum Sensing LuxS Protein and Inhibition of AI-2 Biosynthesis in Aeromonas hydrophila
Source: Molecules. 2018 Oct 12;23(10):2627. doi: 10.3390/molecules23102627 (PMC6222731; doi:10.3390/molecules23102627)
Supplement: Supplementary file 1 [file molecules-23-02627-s001.zip › Supplementary/Supplemntary 11Ramachandran Plot.PDF]

PROCHECK

Ramachandran Plot

2958850

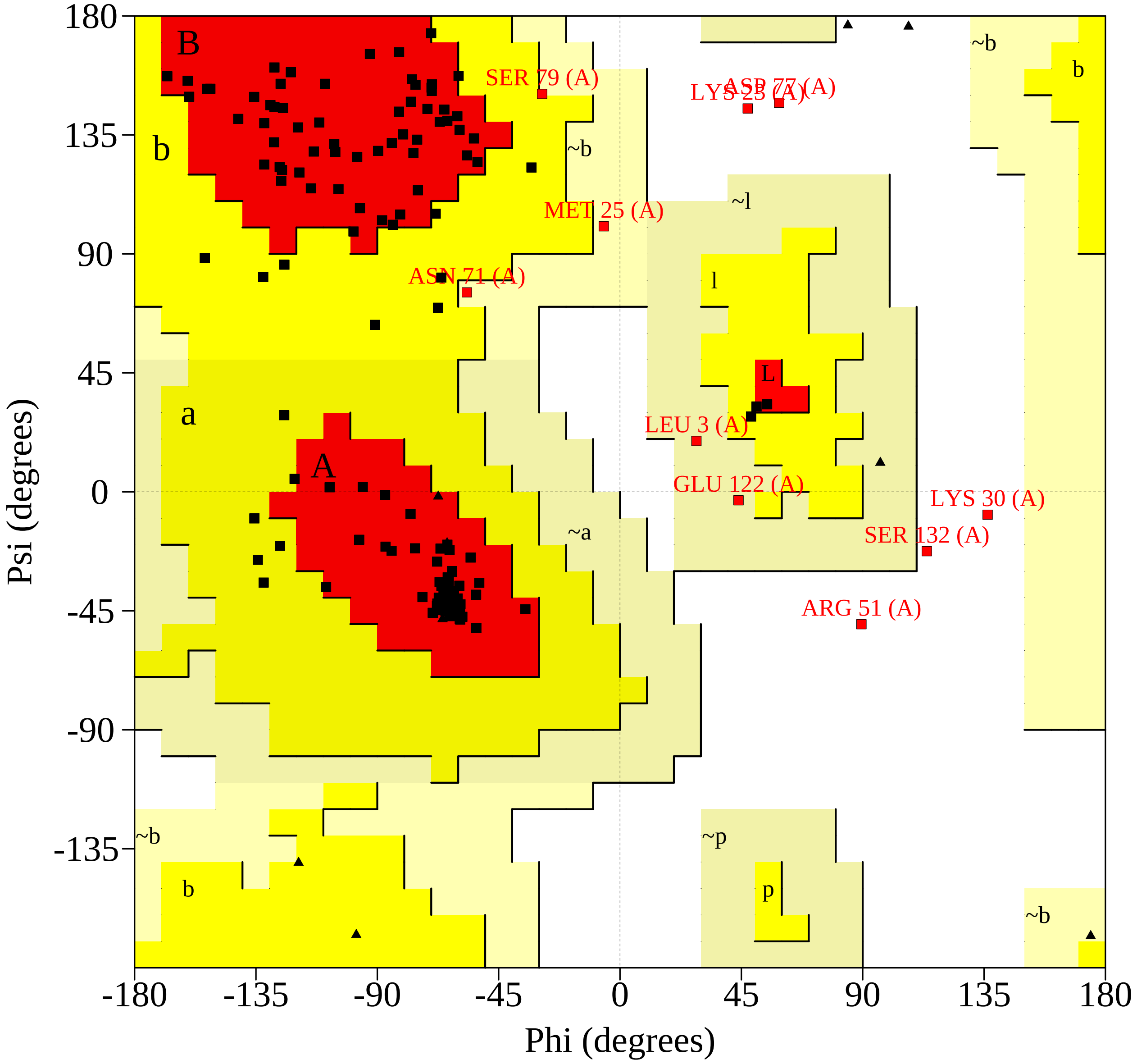

Plot statistics

Residues in most favoured regions [A,B,L]	122	83.0%
Residues in additional allowed regions [a,b,l,p]	15	10.2%
Residues in generously allowed regions [~a,~b,~l,~p]	5	3.4%
Residues in disallowed regions	5	3.4%
	----	------
Number of non-glycine and non-proline residues	147	100.0%
Number of end-residues (excl. Gly and Pro)	2	
Number of glycine residues (shown as triangles)	12	
Number of proline residues	8	

Total number of residues	169	

Based on an analysis of 118 structures of resolution of at least 2.0 Angstroms and R-factor no greater than 20%, a good quality model would be expected to have over 90% in the most favoured regions.
