# Supplementary material for: In-Silico Prediction and Modeling of the Quorum Sensing LuxS Protein and Inhibition of AI-2 Biosynthesis in Aeromonas hydrophila
Source: Molecules. 2018 Oct 12;23(10):2627. doi: 10.3390/molecules23102627 (PMC6222731; doi:10.3390/molecules23102627)
Supplement: Supplementary file 1 [file molecules-23-02627-s001.zip › Supplementary/Supplemntary 2 Model1.pdb_erratluxs.PDF]

Program: ERRAT2
File: /var/www/SAVES/Jobs/95971216//errat.pdb
Chain#:1
Overall quality factor**: 91.925

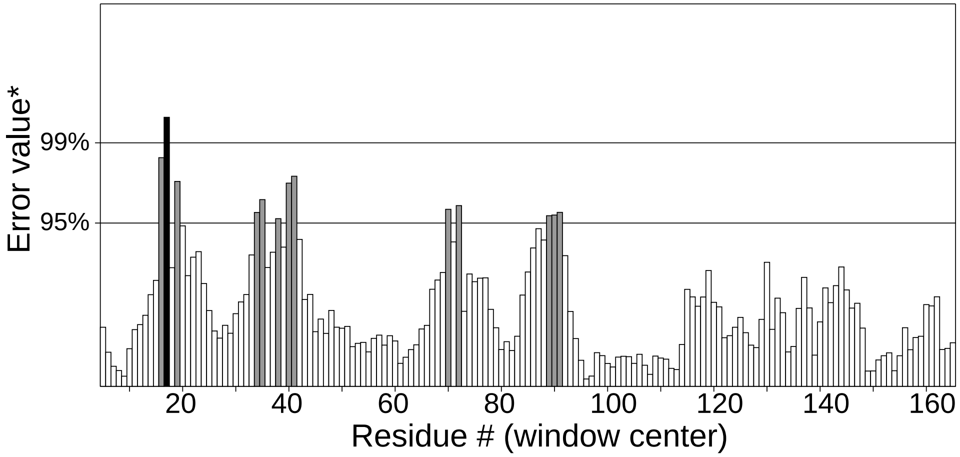

*On the error axis, two lines are drawn to indicate the confidence with which it is possible to reject regions that exceed that error value.

**Expressed as the percentage of the protein for which the calculated error value falls below the 95% rejection limit. Good high resolution structures generally produce values around 95% or higher. For lower resolutions (2.5 to 3A) the average overall quality factor is around 91%.
