# Supplementary figures and images for: In-Silico Prediction and Modeling of the Quorum Sensing LuxS Protein and Inhibition of AI-2 Biosynthesis in Aeromonas hydrophila
Source: Molecules. 2018 Oct 12;23(10):2627. doi: 10.3390/molecules23102627 (PMC6222731; doi:10.3390/molecules23102627)

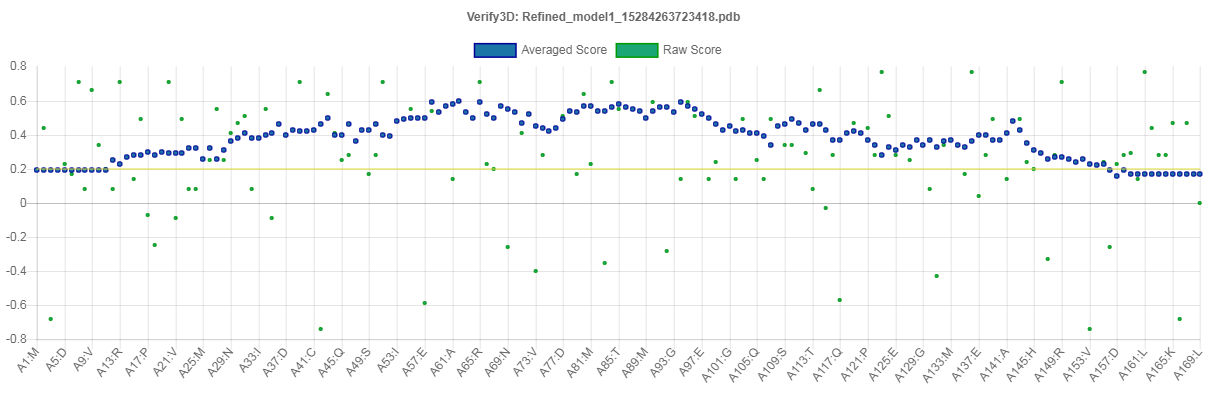

Supplement: Supplementary file 1 [file molecules-23-02627-s001.zip › Supplementary/LuxS model validation verify3d (1).png]
